# Hypoxia tolerance and responses to hypoxic stress during heart and skeletal muscle inflammation in Atlantic salmon (*Salmo salar*)

**DOI:** 10.1371/journal.pone.0181109

**Published:** 2017-07-11

**Authors:** Morten Lund, Maria Krudtaa Dahle, Gerrit Timmerhaus, Marta Alarcon, Mark Powell, Vidar Aspehaug, Espen Rimstad, Sven Martin Jørgensen

**Affiliations:** 1 Section of Immunology, Norwegian Veterinary Institute, Oslo and Harstad, Norway; 2 Nofima AS, Norwegian Institute of Food, Fisheries & Aquaculture Research, Ås, Norway; 3 University of Bergen, Bergen, Norway; 4 Norwegian Institute for Water Research, Bergen, Norway; 5 Department of Food Safety and Infection Biology, Norwegian University of Life Sciences, Oslo, Norway; 6 PatoGen AS, Ålesund, Norway; Fudan University, CHINA

## Abstract

Heart and skeletal muscle inflammation (HSMI) is associated with *Piscine orthoreovirus* (PRV) infection and is an important disease in Atlantic salmon (*Salmo salar)* aquaculture. Since PRV infects erythrocytes and farmed salmon frequently experience environmental hypoxia, the current study examined mutual effects of PRV infection and hypoxia on pathogenesis and fish performance. Furthermore, effects of HSMI on hypoxia tolerance, cardiorespiratory performance and blood oxygen transport were studied. A cohabitation trial including PRV-infected post-smolts exposed to periodic hypoxic stress (4 h of 40% O_2_; PRV-H) at 4, 7 and 10 weeks post-infection (WPI) and infected fish reared under normoxic conditions (PRV) was conducted. Periodic hypoxic stress did not influence infection levels or histopathological changes in the heart. Individual incipient lethal oxygen saturation (ILOS) was examined using a standardized hypoxia challenge test (HCT). At 7 WPI, i.e. peak level of infection, both PRV and PRV-H groups exhibited reduced hypoxia tolerance compared to non-infected fish. Three weeks later (10 WPI), during peak levels of pathological changes, reduced hypoxia tolerance was still observed for the PRV group while PRV-H performed equal to non-infected fish, implying a positive effect of the repeated exposure to hypoxic stress. This was in line with maximum heart rate (*f*_Hmax_) measurements, showing equal performance of PRV-H and non-infected groups, but lower *f*_Hmax_ above 19°C as well as lower temperature optimum (*T*_opt_) for aerobic scope for PRV, suggesting reduced cardiac performance and thermal tolerance. In contrast, the PRV-H group had reduced hemoglobin-oxygen affinity compared to non-infected fish. In conclusion, Atlantic salmon suffering from HSMI have reduced hypoxia tolerance and cardiac performance, which can be improved by preconditioning fish to transient hypoxic stress episodes.

## Introduction

Heart and skeletal muscle inflammation (HSMI) in farmed Atlantic salmon (*Salmo salar*) is an important viral disease in Norwegian aquaculture [1,2]. HSMI has also been reported in farmed Atlantic salmon in Scotland [3], Chile [4] and Canada [5]. The disease is characterized by panmyocarditis (including cellular epicarditis), myocyte necrosis and inflammation in red skeletal muscle [2]. *Piscine orthoreovirus* (PRV) is associated with HSMI [6] and has been detected in farmed salmonids in Ireland, Chile, Japan and Canada [7–13] and in wild salmonids [14]. PRV is non-enveloped with a segmented double-stranded RNA genome, and belongs to the genus *Orthoreovirus* in the family *Reoviridae* [6,15].

Erythrocytes are important target cells for PRV and virus replication in these cells has been confirmed both *in vivo* and *in vitro* [16,17]. Similar to higher vertebrates, piscine erythrocytes secure transport and exchange of oxygen (O_2_) to metabolic active tissues. In contrast to mammals, fish erythrocytes are nucleated with functional organelles like ribosomes and mitochondria, giving them additional properties such as protein synthesis, metabolic activity and immunological functions [18–22]. During PRV infection, high levels of viral transcripts and proteins are detected in the erythrocytes, inducing transcription of genes involved in innate immune responses [17,23]. Hence, one may hypothesize that the viral processing and immune responses may affect hemoglobin production and oxygen binding and transport capacity of infected erythrocytes. This may have consequences for the hypoxia- and stress-tolerance of infected fish. Furthermore, the extensive epicarditis and inflammation of the myocardium associated with HSMI may have additional negative consequences for cardiorespiratory functions and blood transport. Apart from some studies showing reduced cardiac performance to infectious cardiomyopathies in fish [24–26], more knowledge is needed with regards to the secondary cardiorespiratory effects of viral infections, and in particular PRV infection which targets both erythrocytes and cardiac muscle in salmonids.

During their lifespan of 2–3 years, farmed Atlantic salmon encounters several stressful challenges. Intensive rearing conditions (i.e. high stocking density and growth rate) combined with hypoxic episodes, crowding stress and reduced physical condition can be detrimental during viral infections. Naturally occurring acute and chronic hypoxic episodes, ranging from 30–70% oxygen saturation, are described during the seawater production phase [27–29]. Such episodes can be caused by a combination of natural fluctuation in oxygen levels (i.e. alternating water currents, tidal exchange, temperature, net fouling and algae blooms) and variations in oxygen consumption by the fish themselves (i.e. biomass, stress, crowding and behavior) [28,29]. Water temperature and salinity are inversely correlated to the solubility of oxygen and hence affects the consequences of hypoxia in fish. In a cyclic hypoxia trial, Atlantic salmon post-smolts showed reduced appetite and growth when exposed to oxygen saturation lower than 70% at 16°C [30]. Hypoxia during infections represents an additional stressor that may compromise energy resources needed for the immune response. Such mechanisms are poorly studied and need attention as hypoxia clearly affects important physiological functions in fish.

Two important physiological responses to hypoxia in teleost fish are increased cardiac output [31,32] and induced erythropoiesis [33]. PRV infects erythrocytes and induces pathological lesions in cardiac muscle [34]. Heart lesions due to HSMI may affect cardiomyocyte contractility and cardiac function, and hence impair an increase in cardiac output, which may render infected fish less tolerant to environmental hypoxia. Induced erythropoiesis may in turn increase the number of potential target cells for PRV and could therefore affect the development of HSMI. In addition, PRV infection of the erythrocytes may change their ability to transport oxygen.

The aims of this study were to examine whether periodic transient hypoxic stress affects virus levels and disease development during PRV infection, and whether HSMI, alone or in combination with periodic hypoxic stress, affects hypoxia tolerance, cardiorespiratory performance and blood oxygen transport in Atlantic salmon.

## Materials and methods

### Experimental fish and rearing conditions

Seawater adapted Atlantic salmon from the SalmoBreed strain (Bergen, Norway) were used in the study, including N = 705 fish implanted with passive integrated transponder (PIT; Jojo Automasjon AS, Sola, Norway) tags and N = 705 unmarked fish. Tagged fish were i.p. injected with PIT tags two weeks before transfer to the research facility (VESO Vikan, Namsos, Norway). After transfer, fish were acclimatized to brackish water (25‰ salinity) for two weeks before PRV infection. Prior to infection, fish were confirmed negative for PRV, Infectious pancreatic necrosis virus (IPNV) and Salmon pancreas disease virus (SPDV) using quantitative reverse transcription PCR (RT-qPCR). During the infection trial, fish were kept in filtered and UV-radiated brackish water (25‰ salinity), 12°C (±1°C) and continuous light. Fish were fed a standard commercial diet (Skretting AS) using a ratio of 1.5% of total biomass per day, and were starved for 24 h prior to handling and sampling. When the fish were not treated, the biomass in the tanks did not exceed 10 kg/m^3^ and the tank water was provided by a tube overflow system having a flow rate of 7.2 L/min. Before sampling, the fish were euthanized by bath immersion containing benzocaine chloride (200 mg/L water) (Apotekproduksjon AS, Oslo, Norway) for 5 min. The challenge trial was approved by the Norwegian Animal Research Authority and performed in accordance with the recommendations of the current animal welfare regulations: FOR-1996-01-15-23 (Norway).

### Experimental infection trial

The inoculum was pelleted blood cells collected from a previous cohabitation trial, which was the first passage in experimental fish originating from a field outbreak of HSMI in 2012. The preparation of the inoculum is described earlier [35], and was confirmed negative for IPNV, Infectious salmon anemia virus (ISAV), SPDV, Piscine myocarditis virus (PMCV) and Atlantic salmon calicivirus (ASCV) using RT-qPCR [35]. At Day 0, shedder fish (N = 470) were anesthetized by bath immersion for 2–5 minutes in benzocaine chloride (50 mg/L water, Apotekproduksjon AS, Oslo, Norway) and i.p. injected with 0.1 ml of the inoculum, marked by removal of the adipose fin and distributed equally into two 1000 L fiberglass tanks (infected groups), each containing non-infected PIT tagged fish (N = 235 per tank). A third 1000 L fiberglass tank (control group) contained equal numbers of non-infected fish tagged by adipose fin removal (N = 235) and PIT (N = 235). Following anesthesia and inoculation, the i.p. injected fish were transferred to an oxygenated recovery tank before been transferred to the experimental tanks. The fish were continuously monitored until fully recovered.

The infection trial lasted for 15 weeks. Time-points for tests and samplings are displayed in Table 1 and a total overview of the number of fish sampled at each time-point per group and organ is given in S3 Table. Experimental groups are denoted Ctrl (non-infected controls), PRV (infected) and PRV-H (infected and exposed to periodic hypoxic stress, as described in detail below). Sampling for disease development (PRV RNA and histopathological evaluation) was done from all groups before any handling of fish was initiated at 4, 7 and 10 weeks post-infection (WPI). Maximum heart rate and hemoglobin-oxygen affinity measurements were conducted at 10 WPI, when peak levels of pathology were expected. Acute hypoxia challenge test was performed at 4, 7 and 10 WPI. Details on the methods used and sampling procedure performed after each test are described below.

**Table 1:** Experimental groups and sampling time-points.

WPI	0	4			7			10			12			15		
	Day 0	PRV-H	PRV	Ctrl	PRV-H	PRV	Ctrl	PRV-H	PRV	Ctrl	PRV-H	PRV	Ctrl	PRV-H	PRV	Ctrl
Sampling for disease developement	X		x	x	x	x	x	x	x	x	x	x	x	x	x	x
Hypoxia challenge test			x	x	x	x	x	x	x	x						
Heart rate measurement								x	x	x						
Hemoglobin oxygen dissociation curve								x		x						
Periodic hypoxic treatment		x			x			x								

Overview of experimental groups included in the infection trial and weeks post-infection (WPI) at which the specific groups were sampled or tested (indicated by X). “Sampling for disease development” was performed before any test were performed at 4, 7 and 10 WPI. For the other test categories, fish were sampled directly after finishing the respective tests. PRV-H: PRV-infected fish exposed to periodic hypoxic stress, PRV: PRV-infected fish, Ctrl: non-infected fish.

Mortalities during infection were negligible. One fish (0.6%) died in the PRV-H group and two fish (1.2%) died in each of the PRV and control groups.

### Sampling procedures

Organ samples were collected throughout the infection trial from all groups on which physiological tests were performed (Table 1). The number of fish and organ sampled in each group per time-point is specified in S3 Table. Weight and length was registered for sampled individuals and Fulton’s condition factor (k-factor) was calculated (k-factor = weight in grams/(length in cm)^3^ * 100) for each group per treatment and time-point. Twelve fish were sampled prior to PRV cohabitation as negative controls (S3 Table). Heart and gill samples for histopathological evaluation were collected and fixed in 10% phosphate buffered formalin. Within 48 hours after sampling, the formalin was replaced with 70% ethanol and stored at 4°C until further handling.

Two pieces of heart tissue (~2 mm^3^) for RT-qPCR analysis were collected on 1.0 ml tubes (FluidX Ltd., UK) prefilled with 0.5 ml RNA*later™* (Ambion Inc., USA). After euthanasia, blood was collected from the caudal vein using lithium heparin-coated vacutainers (Vacutest klima, Italy) with 20 G Venoject needles. Immediately after sampling, a sub-sample consisting of 20 μl heparinized blood from each individual was added to 1.5 ml Eppendorf tubes prefilled with 1.0 ml Drabkin’s solution (Sigma-Aldrich, USA) for hemoglobin (Hb) analysis. Unless stated otherwise, all samples were stored chilled (5–7°C) and dark until shipment to the laboratory. Tissue samples on RNA*later™* were placed chilled overnight and at -20°C until analyzed.

### RNA isolation and RT-qPCR

From the heparinized blood sample 300 μl was removed and shipped chilled (5–7°C), together with the heart samples on RNA*later™*, to PatoGen Analyse (Ålesund, Norway) for RT-qPCR analysis. PatoGen Analyse performed RNA extraction and RT-qPCR analysis for PRV RNA in heart and heparinized blood. The RT-qPCR assay, validated according to the ISO17025 standard, target PRV transcripts and is described elsewhere [36]. Elongation factor 1α (EF1α) served as an internal reference gene [37] for all RT-qPCR assays performed. Samples having a PRV Ct lower than 37.0 were defined as positive.

### Histopathological analysis

Samples for histopathology were processed and stained with hematoxylin and eosin following standard procedures. The sections from heart and gill were scored blindly and a subsample of hearts (32% of samples scored) was re-scored blindly by a second examinator. Histopathological changes in heart related to HSMI (i.e. epicarditis and myocarditis in the compact and spongy layers) were scored on a continuous visual analogue scale (ranging from 0–3) modified from Mikalsen *et al*. [38]. S1 Table displays the scoring criteria used.

### Periodic hypoxic stress

At 4, 7 and 10 WPI the PRV-H group was exposed to hypoxic stress by reducing oxygen saturation in the tank to 40% O_2_ (±4%) for four hours. The oxygen saturation of 40% was reached within 30 min after gradually reducing the water flow, and kept stable by adjusting the water flow into the tank. The O_2_ level was monitored by two oxygen probes (Handy Polaris, OxyGuard, Denmark) placed 10 cm above the tank floor, one over the tank outlet and one in a sector aside of the outlet. The fish were continuously monitored during the hypoxic exposure and the level of oxygen was recorded manually every 15 min. After ending the hypoxic exposure, the oxygen level was gradually normalized within one hour by increasing the water inlet. The fish were monitored every 15 min for 1–2 hours during the recovery phase after ending the exposure. The biomass in the tank was 39, 37 and 39 kg/m^3^ at 4, 7 and 10 WPI, respectively, during the hypoxic exposure. Water temperature was kept at 12°C during the hypoxic exposure. No mortality occurred during or after the periodic hypoxic stress.

### Acute hypoxia challenge test

Acute hypoxia challenge tests (HCT) were performed in accordance to previous studies [39,40] at 4, 7 and 10 WPI. A water recirculation system with a closed, upright gas equilibrium column was mounted in a separate 1.0 m fiberglass tank containing 425 L of brackish water. Thirty fish from each group were transferred to the HCT tank and allowed to acclimate overnight before the test. Tank biomass was 20.0, 25.7 and 37.2 kg/m^3^ at 4, 7 and 10 WPI, respectively. The HCT was initiated the next morning, consisting of a rapid decrease in oxygen saturation, reaching approximately 25% saturation within one hour, followed by a slower descent of approximately 3–4% saturation per hour until termination of the test. During the test, tank water was pumped (Compact 600, EHEIM, Germany) into the gas equilibrium column containing bio filters, where oxygen was exchanged with nitrogen (N_2_) gas bubbled into the bottom of the column. This ensured a homogenous mixing of N_2_ and water, which was pumped back into the tank and enabled control of ambient oxygen. The flow of N_2_ was controlled by regulating using a controller and solenoid valve (Yara Praxair AS, Oslo, Norway). Oxygen saturation and temperature was measured and logged continuously from two OXROB10 optical oxygen probes in the tank connected to a FireStingO2 oxygen meter (PyroScience GmbH, Aachen, Germany). Oxygen probes were calibrated before each test. When the fish lost the ability to maintain equilibrium (i.e. incipient lethal oxygen saturation (ILOS) was reached), they were quickly removed from the tank and euthanized by a blow to the head. Corresponding time and oxygen level was recorded along with identification of PIT tags to locate group affiliation. The HCT was terminated when all fish had reached ILOS. Samples of heart in RNA*later™* and heparinized blood were collected from N = 10 (4 WPI) and N = 20 (7 and 10 WPI) fish per group. Heart tissue from N = 20 fish per group were sampled at 7 and 10 WPI. As soon as practically possible after sampling, a sub-sample of 20 μl heparinized blood from each individual was added to 1.5 ml Eppendorf tubes prefilled with 1.0 ml Drabkin’s solution for Hb analysis.

### Maximum heart rate measurement

Temperature-dependent maximum heart rate (*f*_Hmax_) was measured at 10 WPI on a subset of fish (N = 16 per group with mean body mass of 170 g) as described by Anttila *et al*. [41]. Briefly, each fish was mildly anesthetized (60 ppm MS-222, buffered to pH 7.0) individually and placed in a small chamber receiving temperature-controlled aerated water (25 ‰ salinity, 12°C) from a Julabo circulating chiller/heater (F32 ME, Julabo GmbH, Seelbach, Germany). An electrocardiogram (ECG) was recorded with a chromel-A measuring electrode positioned lightly on the skin just below the heart and a reference electrode positioned caudal to the heart. The ECG signal was amplified (1000×, Grass P55 amplifier, Astro-Med, Brossard, QC, Canada) and filtered (50 Hz line filter; low-pass: 30 Hz; high-pass: 0.3 kHz) before being stored in a PowerLab data acquisition system (PL3508, PowerLab 8/35, AD Instruments Ltd, Oxford, UK‎). Heart rate was allowed to stabilize for 30 min at 12°C before an intraperitoneal injection of atropine sulphate (2.4 mg kg^-1^ dissolved in 0.9% NaCl; Sigma-Aldrich, Oslo, Norway) that blocked vagal inhibition of the heartbeat. Water temperature was increased in 1°C increments for a cumulative warming rate of 10°C h^-1^, beginning 15 min after the atropine injection. At each temperature increment, both the water temperature and *f*_Hmax_ were stable. *f*_Hmax_ was recorded at each temperature increment by counting R-R intervals for final 15 heartbeats before another temperature increment. The incremental heating was terminated at 20°C, before cardiac arrhythmias were expected. After finishing the test, the fish were euthanized routinely and samples were collected from the heart on formalin as well as heparinized blood from all tested individuals.

### Hemoglobin-oxygen dissociation measurement

Hemoglobin-oxygen dissociation measurements were analyzed according to the Tucker method [42]. Measurements were performed on blood sampled from the PRV-H (N = 12) and control group (N = 12) at 10 WPI. Fish were euthanized routinely as described above and heparinized blood (1.5–2.0 ml) was drawn from the caudal vein. Individual samples measuring less than 2.0 ml were pooled with the sample from a second individual within the same group. A total amount of 2.0–2.5 ml blood was transferred into a rotating blood tonometer (handmade in glass) and gassed with air or N_2_ gas. The blood was incubated with 1.0 × 10^−5^ M propranolol (Actavis, Iceland) in phosphate buffered saline to block the effects of adrenaline and nor-adrenaline on the β-adrenoreceptors, affecting oxygen binding affinity of salmonid hemoglobin [43]. Within the tonometer, the sample was kept at a temperature of 11–13°C by circulating chilled water around the tonometer. Successive desaturation of the blood was undertaken by gassing the blood sample with nitrogen. At successive levels of oxygenation determined by direct measurement of blood PO_2_, the oxygen content of the blood was determined using the Tucker method in a thermostatted TC500 Tucker Cell (Strathkelvin Instruments, Scotland). The procedure was carried out on each blood sample to generate data from at least 5–7 different levels of oxygenation. The PO_2_ levels were coupled with the spectrophotometric measured hemoglobin concentration of the sample. After finishing the measurements of the individual sample, 20 μl of the heparinized blood was added to 1.5 ml Eppendorf tubes prefilled with 1.0 ml Drabkin’s solution for Hb measurement, as described below.

### Hemoglobin measurement

Hemoglobin concentration (g/100 ml blood) was measured spectrophotometrically as cyanmethemoglobin on heparinized blood samples mixed 1:500 with Drabkin’s solution by determining the absorbance at 540 nm. The procedure was performed on a TECAN Sunrise microplate reader (TECAN, Switzerland) according to the manufacturer’s procedure for Drabkin’s reagent (Cat.no. D5941, Sigma-Aldrich, Scotland). The Hb concentration was determined from a standard curve prepared from bovine Hb powder (H2500-1G, Sigma-Aldrich, Scotland).

### ATP measurement

Adenosine triphosphate (ATP) concentration (nmol/μl blood) was measured on heparinized blood samples (100 μl) snap frozen on liquid nitrogen. The ATP concentration was calculated by fluorometric detection of ATP using a colorimetric/fluorometric assay kit (Cat.no. MAK190, Sigma-Aldrich, Scotland) on an EnSpire 2300 Multilable plate reader (PerkinElmer, USA) according to the manufacturer’s protocol. The amount of ATP in each well was obtained from the standard curve and the ATP concentration in each sample was calculated as described by the manufacturer.

### Data analysis and statistics

The statistical analyses and plotting were performed in R (version 3.3.1) for all data. Differences in viral RNA levels, ILOS, histopathological changes in heart, hemoglobin and ATP levels between the groups were examined using the non-parametric Mann-Whitney unpaired rank test. An unpaired Student’s t-test was used to examine differences in k-factor and weight. A linear regression analysis and Pearson correlation analysis (R; stats and corrplot packages) was performed on individual weight and oxygen saturation levels from data collected during the HCT’s (both compiled and separately). Kaplan-Meier plots were generated from the HCT by using the additional “survival” package in R. Differences between the curves were tested by performing a Peto & Peto modification of the Gehan-Wilcoxon test embedded in the same package. The data from the temperature dependent heart rate measurement was computed by using the following additional packages: RColorBrewer, devtools, AquaR (github "gtimmerhaus/aquaR")).

An oxygen dissociation curve was fitted by a local polynomial regression fit (“loess” function in the “stats” package) through O_2_ saturation and PO_2_ registrations. Whole blood oxygen content was calculated according to Tucker [40]. From these measurements, data was corrected for Hb concentration after subtracting the physically dissolved O_2_ according to [44] and the percent saturation of Hb calculated. Data was then log transformed (log10((O_2_/gHb)/(1-(O_2_/gHb)) and log10 PO_2_) according to [45] and a linear regression line was fitted through the linearized data. The K_d_ (zero intercept) and Hill coefficient (n_H_) was determined using SigmaPlot 10.0 (Systat Software Inc, London, UK). The inverse log of the K_d_ is equivalent to the P_50_. A *p*-value < 0.05 was considered statistically significant for all data analyzed.

A Pearson correlation and linear regression analysis were performed (R; stats and corrplot packages) for associations between PRV Ct values in heart and blood and Hb concentration.

## Results

### Body metrics

From the start until termination of the infection trial (15 WPI), mean body weight increased (from 76.7±2.5 g to 258.1±9.5 g, N = 12 and N = 20) and mean condition factor remained unchanged (from 1.26±0.02 to 1.24±0.02, N = 12 and N = 20) for the non-infected group (S1 Fig, S3 Fig). In the infected group, the mean body weight and condition factor at 15 WPI was 268.9±9.8 g and 1.26±0.02 (N = 20), respectively (S1 Fig, S3 Fig). There were no significant differences in body weight or condition factor between infected groups (PRV versus PRV-H group) at any time-points. However, compared to the Ctrl group, PRV-H had significantly lower body weight (*p* < 0.05) and condition factor (*p* < 0.01) at 12 WPI, and the PRV group had significantly lower weight (*p* < 0.05) at 10 WPI and condition factor at 10 (*p* < 0.05) and 12 WPI (*p* < 0.01) (S1 Fig, S2 Fig). No significant differences in body weight or condition factor were detected between any groups at 15 WPI (S1 Fig, S2 Fig). Body weight of PRV and PRV-H fish included in the HCT was significantly higher compared to the Ctrl group at 4 WPI but not at the later time-points (S3 Fig). Regression and Pearson correlation analyses of data recorded from all HCTs or at each time-point separately, showed no effects of individual body weight on oxygen saturation at ILOS (r^2^ = 0.05) (S3 Fig).

### Periodic hypoxic stress does not affect PRV RNA levels or HSMI development

**Fig 1:**
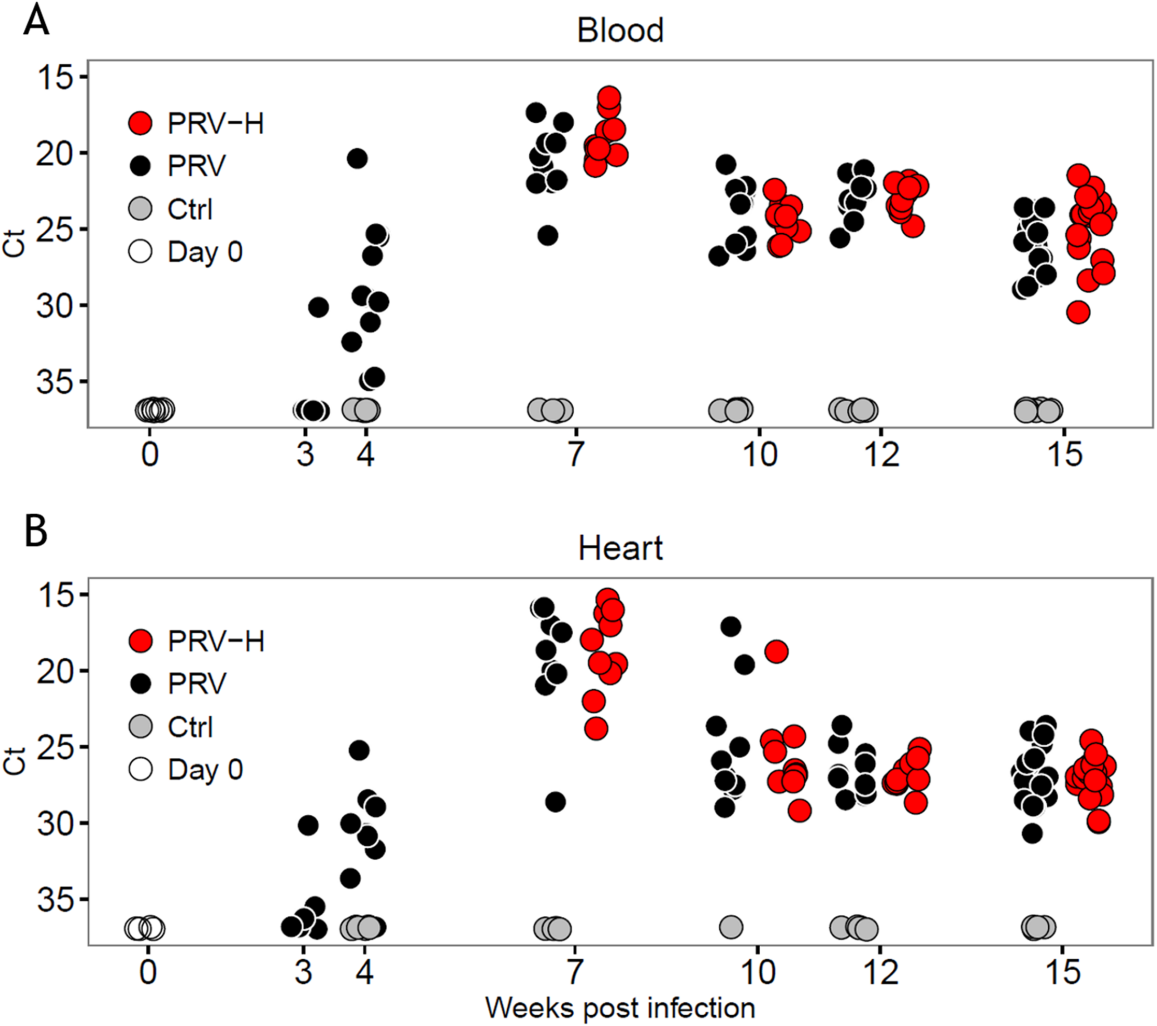
PRV RNA levels in blood and heart. PRV RNA levels (Ct values) in blood (A) and heart (B) from naïve fish sampled at Day 0 (white dots), non-infected controls (Ctrl, grey dots), PRV-infected (PRV, black dots) and PRV-infected fish exposed to periodic hypoxic stress (PRV-H, red dots), at each time-point during the infection trial. Weeks post-infection (WPI) are indicated on the x-axis. Ct value ≥ 37.0 indicates no virus RNA detected. A non-parametric Mann-Whitney unpaired rank test was performed between the groups at all time-points detecting no significant differences between the groups (*p* > 0.05).

**Fig 2:**
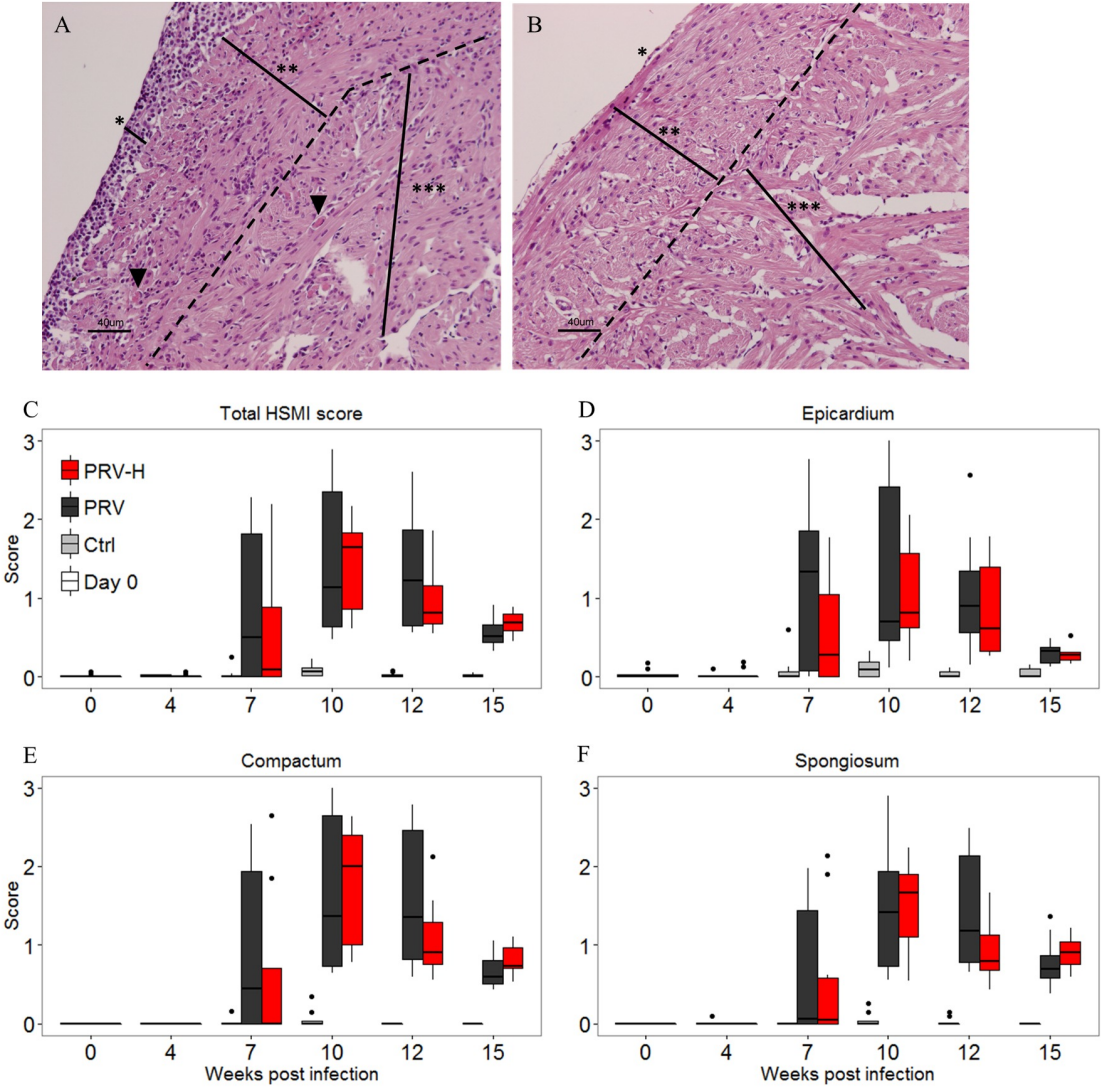
Histological scoring of inflammatory changes in the heart. A. Section of heart tissue (hematoxylin and eosin staining) showing epicarditis and myocarditis from a heart having an average cardiac inflammation score of 1.86. The PRV RNA Ct value in heart and blood of the fish in A was 17.2 and 20.5, respectively. B. Picture of a non-infected heart. The hearts in A and B were sampled 7 weeks post infection. *, ** and *** indicate epicardium, compactum and spongiosum, respectively. Arrowhead indicate myocardial necrosis. Bars indicate the approximate thickness of the respective layers. 20x magnification in A and B. C—E. Development of inflammatory changes is displayed for each group throughout the challenge trial. Ctrl: non-infected controls, PRV: PRV-infected fish, PRV-H: PRV-infected fish exposed to periodic hypoxic stress. Inflammatory changes in epicardium, compactum and spongiosum were scored from sections of the heart ventricle using a continuous visual analogue scale ranging from 0 (no inflammatory changes) to 3 (severe panmyocarditis). The total HSMI score was calculated from the mean of scores from the separate heart compartments. The lower and upper border of boxes indicates the 25^th^ and 75^th^ percentiles, respectively and the centerline indicates the 50^th^ percentile. The upper and lower whiskers correspond to the highest and lowest value of the 1.5*IQR (inter-quartile range). A non-parametric Mann-Whitney unpaired rank test was performed between the groups at all time-points. Weeks post-infection (WPI) are indicated on the x-axis.

In order to evaluate the effects of three short-term hypoxic exposure (40% O_2_ for 4 h) on HSMI pathogenesis, virus RNA levels and histopathological changes were evaluated in PRV-H and PRV groups at 4, 7, 10, 12 and 15 WPI (Figs 1 and 2). Non-infected controls were negative for virus and pathological changes at all time-points. There were no differences in virus RNA levels and histopathology scores between groups prior to the first hypoxia exposure (4 WPI). After hypoxic stress, there were no significant differences in virus RNA levels in blood and heart between the PRV-H and PRV groups at any time-point (Fig 1, data in S2 Table).

Histopathological changes consistent with HSMI [46,47] including epicarditis, myocardial mononuclear cell infiltration and myocardial necrosis were detected from 7 WPI in both infected groups (Fig 2A and 2B). Pathological changes were scored on a continuous visual analogue scale separately in three compartments of the heart (i.e. epicardium, compactum and the spongious (trabecular) layers). The averaged sum of scores for all compartments were calculated as the total HSMI score (Fig 2C). In both groups, total HSMI score peaked at 10 WPI and declined until 15 WPI. There were no significant differences in either total HSMI score or separate scores in each compartment between PRV and PRV-H groups (Fig 2C–2E). However, the PRV-H group tended to have a stronger reduction in total score levels and compactum and spongiosum scores from peak levels (10 WPI) until 12 WPI compared to the PRV group (Fig 2C, 2D and 2E).

### Hypoxia tolerance is reduced during HSMI and improved by repeated hypoxic stress

**Fig 3:**
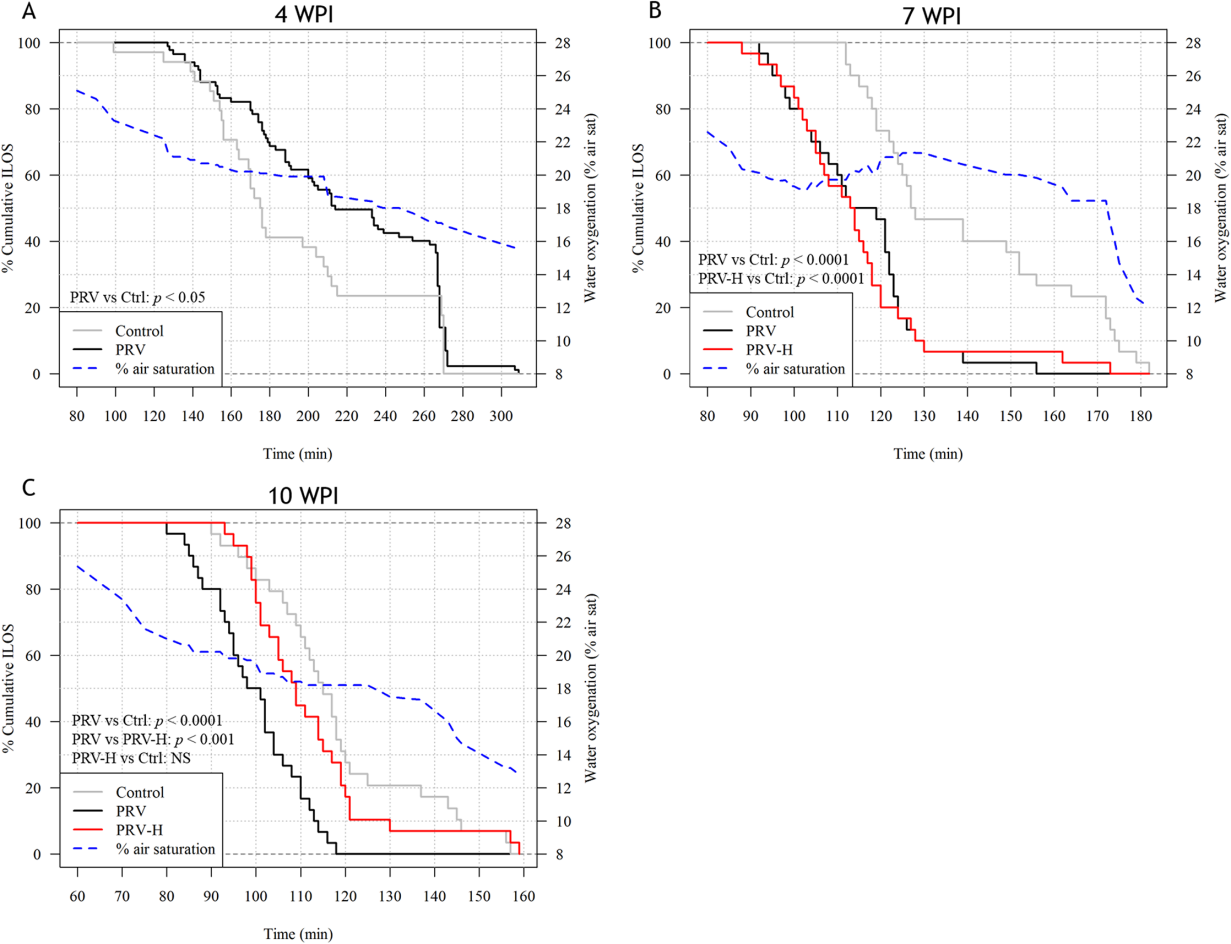
Kaplan-Meier plots of tolerance time during hypoxia challenge test. Percent cumulative incipient lethal oxygen saturation (ILOS) levels over time during acute hypoxia challenge of the Ctrl (grey line), PRV (black line) and PRV-H (red line) groups at 4 (A), 7 (B) and 10 (C) weeks post-infection (WPI). Secondary y-axis and dotted line (blue) shows water oxygen levels (% air saturation). Statistical significance levels are indicated in the bottom left of each plot after performing a Peto & Peto modification of the Gehan-Wilcoxon test between the curves for each group; Ctrl vs PRV (4, 7 and 10 WPI), Ctrl vs PRV-H (7 and 10 WPI) and PRV-H vs PRV (10 WPI). NS indicates not significant.

To test the hypothesis that PRV-infection and HSMI-related cardiomyopathy affects hypoxia tolerance, an acute hypoxia challenge test (HCT) [48] was performed in common garden experiments including 30 fish from each group at 4, 7 and 10 WPI. Time-course of water oxygenation and temperature for each HCT are shown in S4 Fig. Cumulative incipient lethal oxygen saturation (ILOS) levels for each group and time-point are plotted as Kaplan-Meier (K-M) duration curves (Fig 3). Average values for ILOS and time (minutes) to cull 50% of the test groups (T_50_) at each time-point are displayed in Table 2.

**Table 2:** Measures from the hypoxia challenge test.

	4 WPI		7 WPI		10 WPI	
	*ILOS*	*T*_*50*_	*ILOS*	*T*_*50*_	*ILOS*	*T*_*50*_
**Ctrl**	20.32±0.17^a^	175	19.26±0.61^a^	127	17.86±0.33^a^	115
**PRV**	19.74±0.16^a^	222	20.41±0.15^a^	113	19.32±0.16^b^	98
**PRV-H**			19.99±0.21^a^	113	18.36±0.30^a^	109

Average ILOS levels (± SE) and time (min) to cull 50% of the experimental population (T_50_) for each group (N = 30) during HCT performed at 4, 7 and 10 weeks post-infection (WPI). Groups are non-infected controls (Ctrl), infected (PRV) and infected exposed to periodic hypoxic stress (PRV-H). Statistical differences (*p* < 0.05) are indicated by superscript letters.

**Fig 4:**
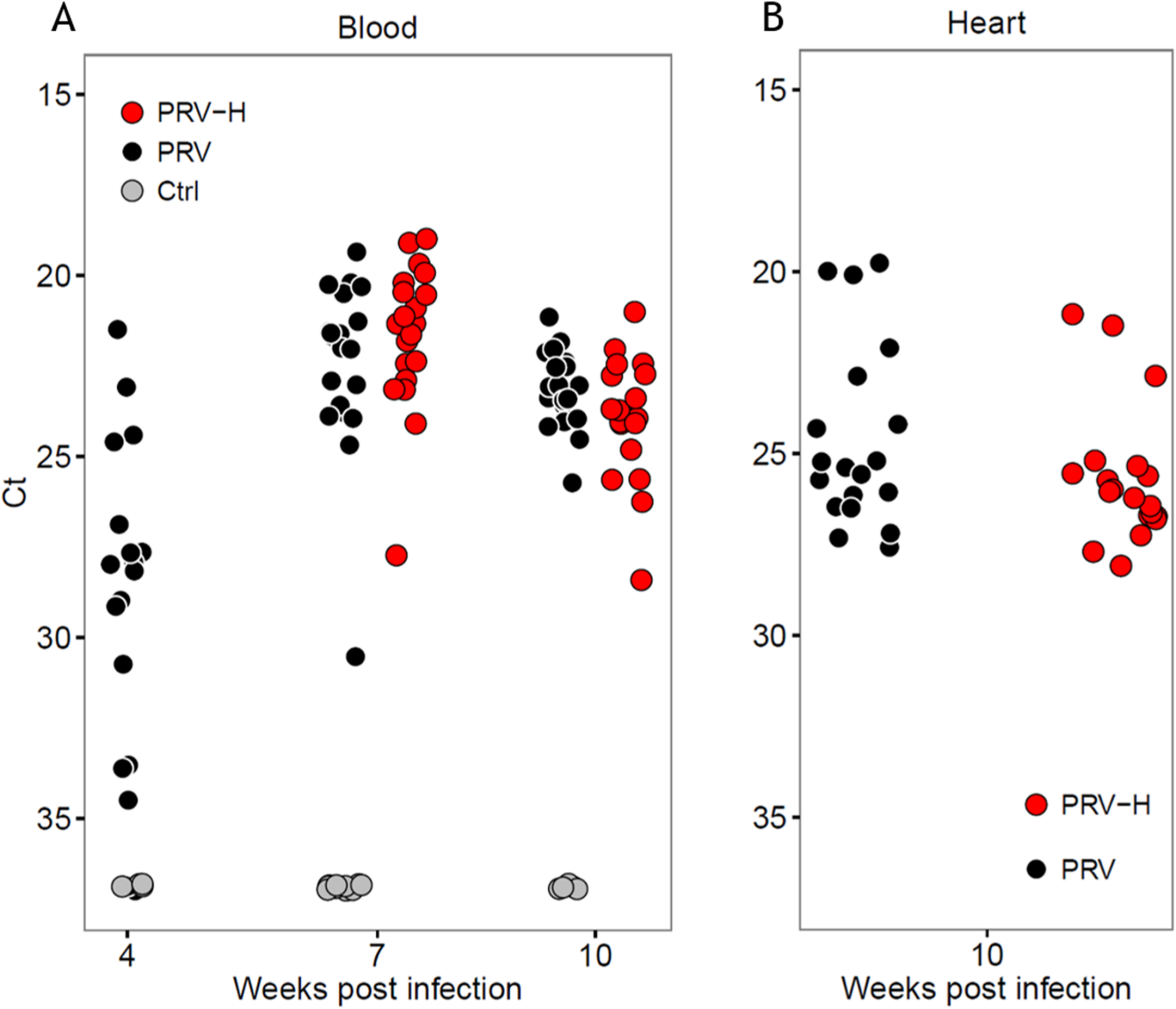
PRV RNA levels–HCT. PRV RNA levels (Ct values) in blood (A) and heart (B) from fish included in the HCT. Groups are non-infected controls (Ctrl, grey dots), infected (PRV, black dots) and infected exposed to periodic hypoxic stress (PRV-H, red dots). A non-parametric Mann-Whitney unpaired rank test was performed between the groups at all time-points detecting no significant differences between the groups (*p* > 0.05). Weeks post-infection (WPI) are indicated on the x-axis.

At 4 WPI (prior to first hypoxic exposure), there were no group differences with regard to average ILOS levels (Table 2). However, infected fish were more hypoxia tolerant than Ctrl according to K-M curves (*p* < 0.05; Fig 3A) and T_50_ values (222 versus 175, respectively; Table 2) at 4 WPI, but due to weight differences between the groups at this time point, an effect of infection could not be confirmed. Despite considerable differences in PRV RNA Ct values at 4 WPI, no correlation between Ct values in blood and hypoxia tolerance was found in infected fish (*p* > 0.05, unpublished data). At 7 WPI, when PRV RNA levels peaked and heart pathology was pronounced, both infected groups were less hypoxia tolerant than Ctrl according to K-M curves (*p* < 0.0001; Fig 3B) and T_50_ values (113 versus 127; Table 2). Average ILOS for PRV and PRV-H groups were not significantly different from, but numerically higher than the Ctrl group (Table 2). At 10 WPI, when heart pathology reached peak levels, K-M curves showed that the PRV group was less hypoxia tolerant compared to both the Ctrl (*p* < 0.0001) and the PRV-H (*p* < 0.001) group (Fig 3C). ILOS levels were higher and T_50_ values were decreased for the PRV group (19.32 / 98) compared to the Ctrl (17.86 / 115) and the PRV-H (18.36 / 109; Table 2) group. There were no differences in the PRV RNA levels in blood and heart between infected individuals selected for the HCT at any time-point (Fig 4).

**Fig 5:**
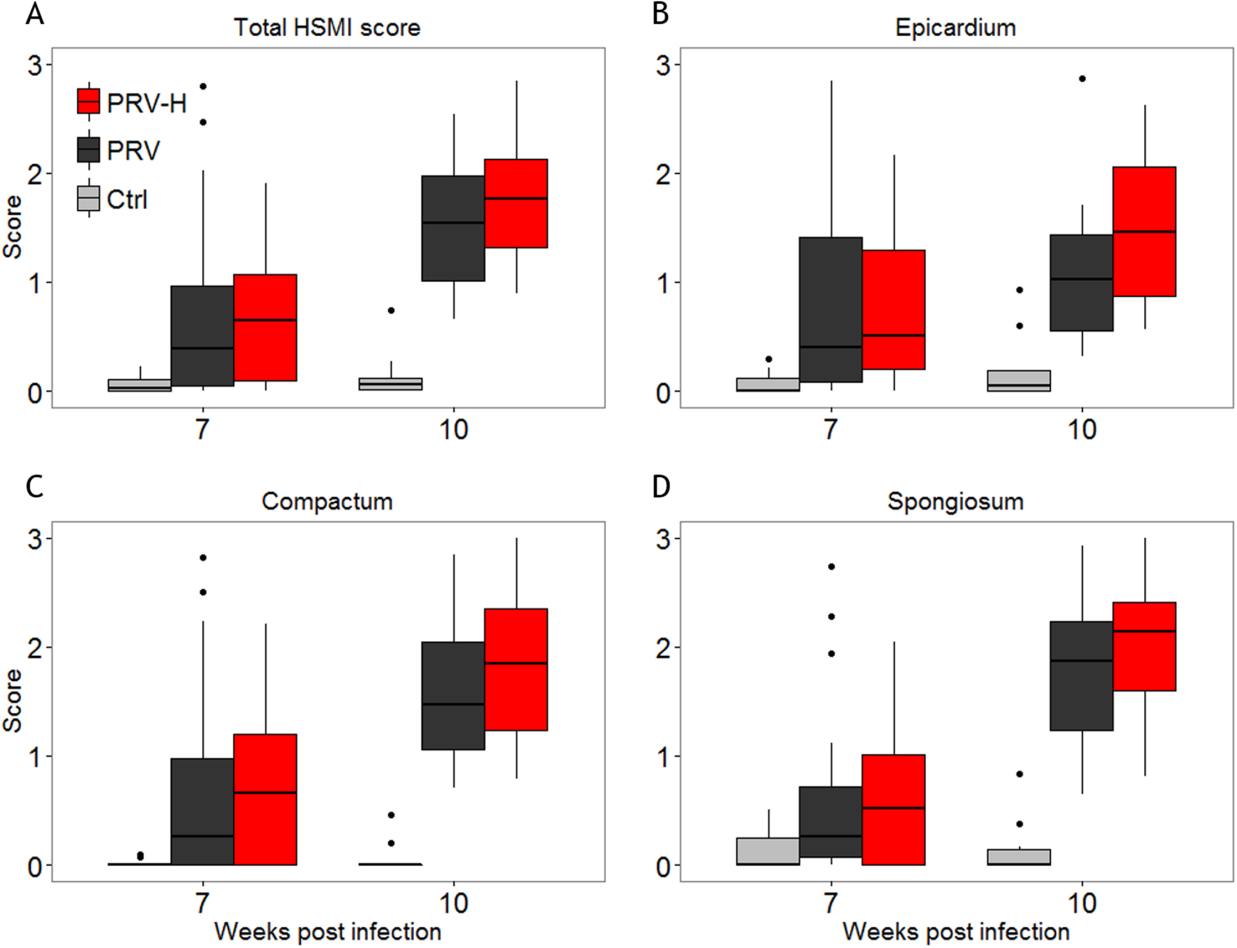
Histological scoring of inflammatory changes in the heart–HCT. Scoring of the inflammatory changes for the fish included in the HCT are displayed by groups. Ctrl: non-infected control group, PRV: PRV-infected group, PRV-H: PRV-infected exposed to periodic hypoxic stress. Inflammatory changes in epicardium, compactum and spongiosum were scored from sections of the heart ventricle using a continuous visual analogue scale ranging from 0–3. The total HSMI score was calculated from the mean of scores from the separate heart compartments. The lower and upper border of boxes indicates the 25^th^ and 75^th^ percentiles, respectively and the centerline indicates the 50^th^ percentile. The upper and lower whiskers correspond to the highest and lowest value of the 1.5*IQR (inter-quartile range). A non-parametric Mann-Whitney unpaired rank test was performed between the groups at all time-points detecting no significant differences between the groups (*p* > 0.05). Weeks post-infection (WPI) are indicated on the x-axis.

The histopathological scoring showed a trend of higher inflammation score in all heart compartments for the PRV-H group compared to the PRV-infected group (Fig 5). Histopathological evaluation of gill sections collected from all fish included in the HCT showed no pathological lesions (data not shown).

### HSMI-related cardiomyopathy reduces optimum temperature (*T*_opt_) for aerobic scope, which is improved after repeated hypoxic stress

**Fig 6:**
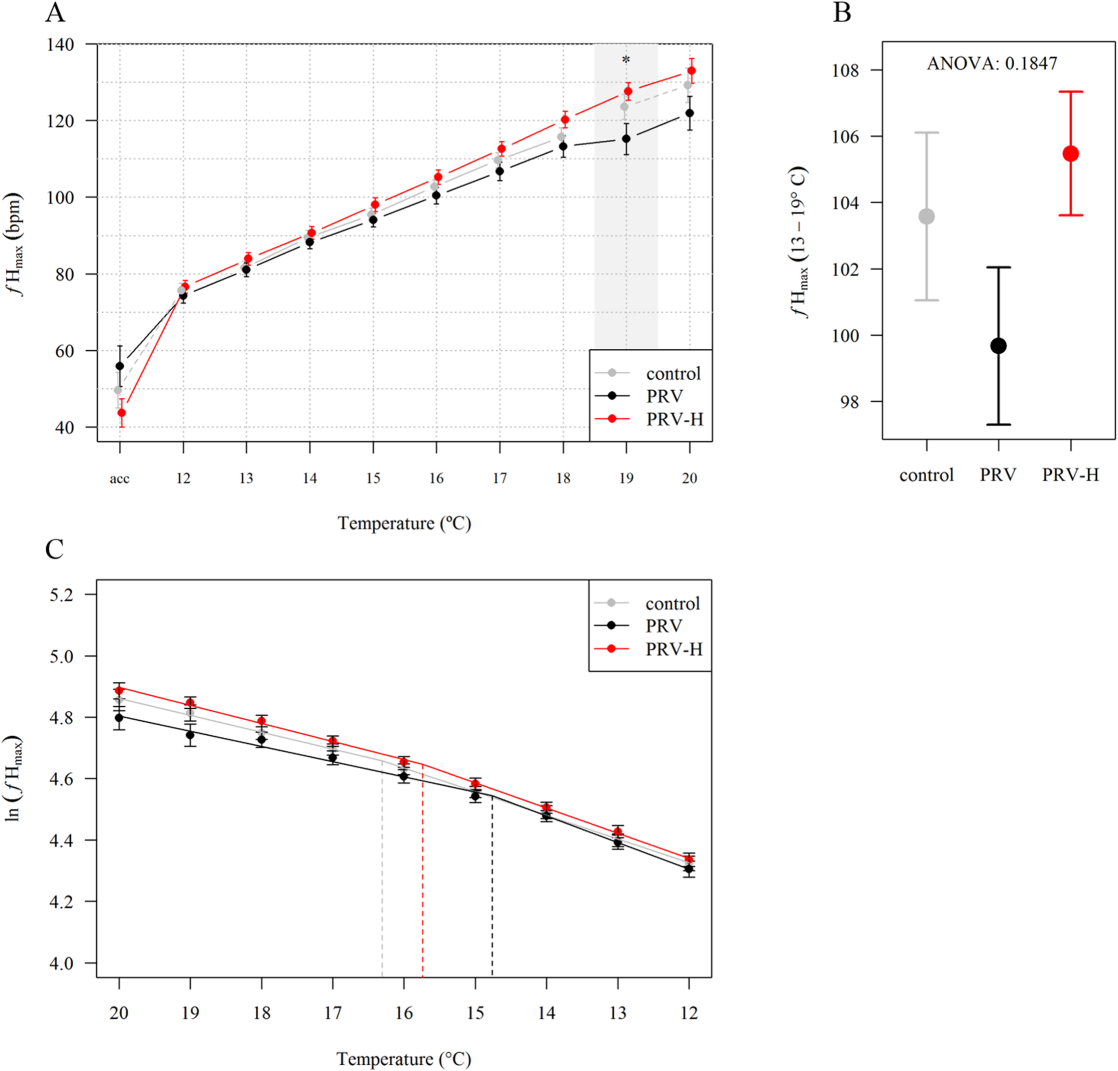
Maximum heart rate measurements. Maximum heart rate (*f*_Hmax_) measurements at 10 WPI in non-infected controls (Ctrl, grey line), PRV-infected (PRV, black line) and PRV-infected exposed to periodic hypoxic stress (PRV-H, red line). A: Average *f*_Hmax_ (±SE) during temperature increase for each group. Dashed lines between dots indicate that half or more individuals of the initial population were missing or had cardiac arrhythmia and therefore were removed from the measurement. Grey areas and asterisks indicate significant differences between groups (ANOVA, *: *p* < 0.05). Point "acc" shows *f*_Hmax_ after acclimation at 12°C, just before atropine injection. B: Average *f*_Hmax_ (±SE) of the three groups between 13 and 19°C. C: T_opt_ for aerobic scope calculated from Arrhenius breakpoint temperature of *f*_Hmax_ for each group.

In order to evaluate if cardiac histopathological changes associated with HSMI and additional periodic hypoxic stress affect specific measures of cardiac performance and thermal tolerance, maximum heart rate (*f*_Hmax_) measurements were performed at 10 WPI, when histopathological changes were most pronounced. Infected fish included in the analysis had similar levels of histopathological changes in the heart and no statistical differences were detected (data in S5 Fig). There were no group differences in *f*_Hmax_ during the linear phase (12–18°C), but PRV-infected fish had lower *f*_Hmax_ at 19°C (Fig 6A). Average *f*_Hmax_ between 13–19°C showed that the PRV group had respectively 5.7 and 6.1 lower *f*_Hmax_ than the PRV-H and Ctrl (*p* > 0.05) groups (Fig 6B). A two-way ANOVA showed that the PRV group had a lower heart rate (-4.7 BPM, *p* = 0.07) than the other two groups (Fig 6B). *T*_opt_ for aerobic scope was determined from Arrhenius breakpoint temperature calculations for *f*_Hmax_ (Fig 6C), showing a higher *T*_opt_ for the Ctrl (16°C) group compared to the PRV-H (15.3°C) and the PRV (14.7°C) groups.

No significant correlation (Pearson’s product-moment correlation) between heart rate and virus RNA level in blood was found (r = 0.15, *p* = 0.51) (data in S6 Fig). The linear model indicated a decreased *f*_Hmax_ with increasing virus RNA level. However, this effect was weak with 0.89 decrease in *f*_Hmax_ per virus Ct value (i.e. doubling of virus RNA transcripts) (data in S6 Fig).

### PRV infection combined with periodic hypoxic stress reduces blood oxygen affinity

**Fig 7:**
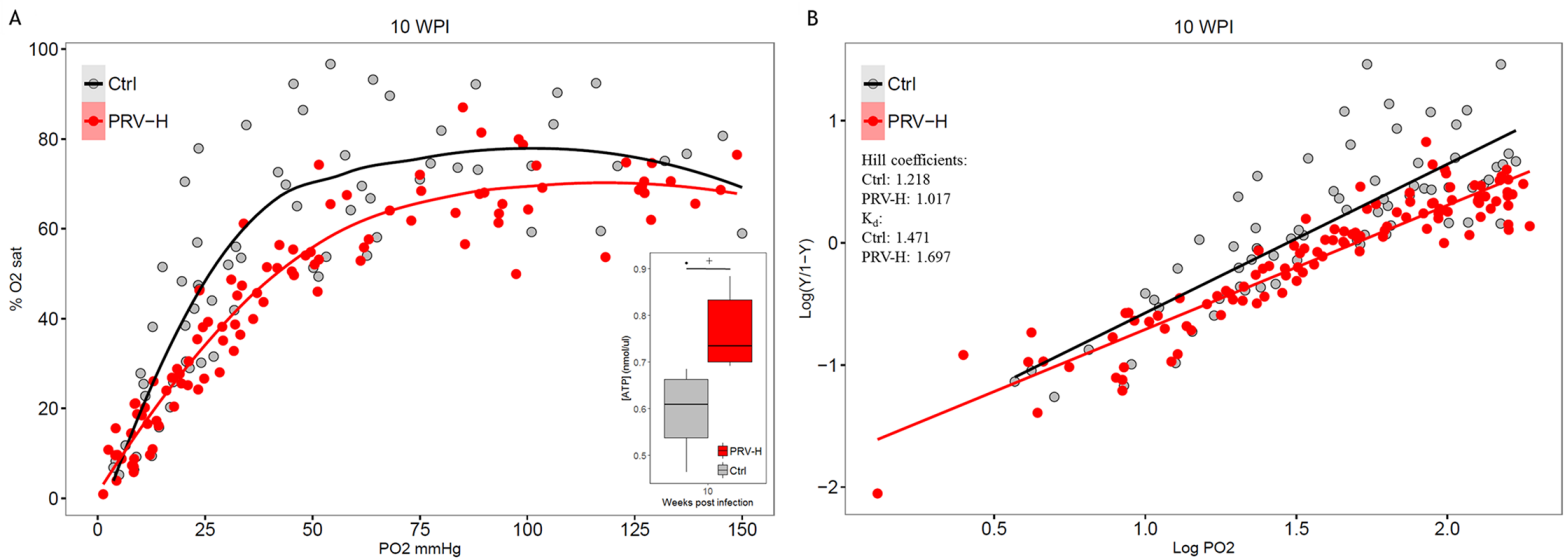
Blood oxygen binding affinity. Blood oxygen binding affinity in PRV-infected fish exposed to periodic hypoxic stress (PRV-H, red curve/line) and non-infected controls (Ctrl, black curve/line) at 10 weeks post-infection (WPI). A: Hb-O_2_ dissociation curves (ODC) relating partial pressure of oxygen (P_O2_; x-axis) with Hb-oxygen saturation (y-axis). ATP concentrations are shown in inset with significance level (*p* = 0.07, indicated by +) according to non-parametric Mann-Whitney unpaired rank test. B: Linear regression of ODC from log-transformed data showing Hill coefficients and K_d_ (zero intercept) values for the groups.

The effect of PRV-infection and periodic hypoxic stress on blood oxygen affinity was evaluated from Hb-oxygen dissociation curves (ODC) calculated for PRV-H and Ctrl groups at 10 WPI (Fig 7). Measurements were corrected for Hb concentrations. Average Hb levels for Ctrl and PRV-H at 10 WPI were respectively 8.0 and 9.6 g/100 ml in the fish sampled for the ODC (*p* = 0.06), and average ATP levels 0.63 and 0.74 nmol/μl (*p* = 0.07; Fig 7A, data in S7 Fig). There was an apparent right-shift of the ODC and resulting increased P_50_ for the PRV-H group (49.7 mm Hg) compared with the Ctrl group (29.5 mm Hg) (Fig 7A). Maximal oxygen saturation was also lower in the PRV-H group compared with the controls (Fig 7A). Linear regression analysis of ODC data also showed a higher Hill coefficient and K_d_ value for the Ctrl versus the PRV-H group (Fig 7B).

**Fig 8:**
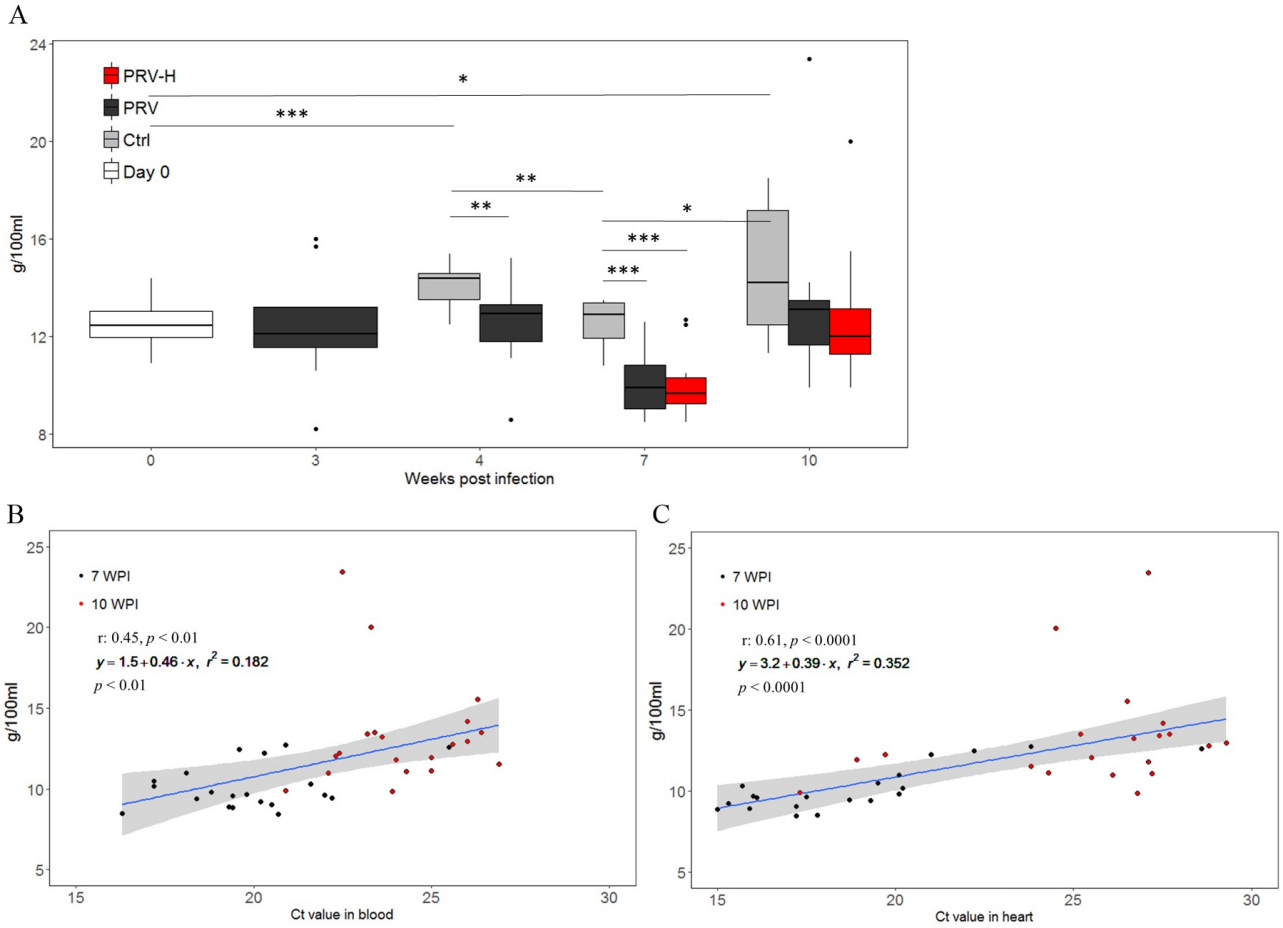
Hemoglobin levels and correlations with PRV Ct values in fish sampled for disease development. A: Hemoglobin (Hb) concentration (y-axis) in non-infected controls (Ctrl, grey), PRV-infected (PRV, black) and PRV-infected exposed to periodic hypoxic stress (PRV-H, red). The lower and upper border of boxes indicates the 25^th^ and 75^th^ percentiles, respectively and the centerline indicates the 50^th^ percentile. The upper and lower whiskers correspond to the highest and lowest value of the 1.5*IQR (inter-quartile range). A student t-test was performed to test differences in Hb values between the groups. Significance is indicated by *, ** and *** with a *p* < 0.05, 0.01 and 0.001, respectively. B and C: Pearson correlation analysis and linear regression analysis of PRV Ct values (x-axis) in blood (B) and heart (C) and hemoglobin concentrations (y-axis). The analysis was performed on merged data-points from 7 (black dots) and 10 (red dots) weeks post infection (WPI). The correlation coefficient (r) with *p* value and linear regression output with the regression line in blue (95% CI is shaded gray) and *p* values is stated.

The hemoglobin concentration in the fish sampled for disease development (S3 Table) was significantly higher in the Ctrl group at 4 and 10 WPI compared to Day 0 (*p* < 0.001 and *p* < 0.05) (Fig 8A). Furthermore, the Hb concentration in the Ctrl group was significantly higher at 4 and 10 WPI compared to 7 WPI (*p* < 0.01 and *p* < 0.05) (Fig 8A). At 4 and 7 WPI, the Hb concentration was significantly higher in the Ctrl group at 4 and 7 WPI compared to the PRV-infected groups (*p* < 0.01 and *p* < 0.001) (Fig 8A). Pearson correlation analysis showed positive correlation between PRV Ct values and Hb concentration in blood (r = 0.45, *p* < 0.01) and heart (r = 0.61, *p* < 0.001) in the compiled data from 7 and 10 WPI in the fish sampled for disease development (N = 40, Ctrl group omitted) (Fig 8B and 8C). A linear regression analysis showed that for every increase in Hb concentration in blood, the Ct value of PRV RNA in blood and heart increases with 0.46 and 0.39 (adj. r^2^ = 0.18 and 0.35, *p* < 0.01 and *p* < 0.0001), respectively (Fig 8B and 8C, statistics in S8 Fig).

## Discussion

In the present study, we found that both hypoxia tolerance and cardiac performance are impaired during peak levels of PRV-infection and HSMI lesions in Atlantic salmon. Repeated short-term hypoxic exposure during disease development diminished these effects.

The hypoxia tolerance challenge test was performed at early viral replication prior to HSMI specific lesions (4 WPI), peak virus load and initial lesions (7 WPI), and peak levels of lesions (10 WPI). Contrary to our expectations, the HCT performed at 4 WPI showed a significantly higher hypoxia tolerance for the infected group compared to the controls. The PRV-infected group had also significantly higher weight compared to the Ctrl group. Increased body mass has variable effect on hypoxia tolerance in fish [48–50], however we found no effect of body weight on ILOS, either across all time-points (all data compiled) or at separate time-points. Furthermore, at 4 WPI, 70% of the infected individuals had moderate levels of viral RNA in blood cells. The improved hypoxia tolerance could be related to a priming response caused by the infection in these cells. For example, a recent study by Johansen et al. [51] showed upregulation of genes related to iron metabolism and erythropoiesis four weeks after PRV infection. However, this should be reflected by increased levels of hemoglobin, which was not found here. In a similar PRV study, induced erythrocytic transcription of catalase, glucocorticoid receptor and hypoxia-inducible transcription factor (HIF-1α2) was observed [23]. These genes have important roles in responses to tissue hypoxia in higher vertebrates [52,53]. Hence, PRV-infection of erythrocytes induces genes that may increase the hypoxia tolerance.

At 7 WPI, when PRV RNA levels peaked and histopathological lesions were developing, the PRV-infected groups were less hypoxia tolerant compared to non-infected controls. Previously, the transcription of innate antiviral genes are shown to be strongly induced at this stage of the PRV-infection [17,23,51]. The viral transcription, subsequent innate antiviral immune response in erythrocytes and the onset of myocardial inflammation may contribute to the impaired hypoxia tolerance. The group exposed to periodic hypoxic stress (PRV-H; 4 h of 40% O_2_) intended to simulate incidences of hypoxia in the field, i.e. during handling operations or natural reduction in water exchange during the turn of tidal currents [28,54], did not show any difference in performance from the PRV group. This implies that a single previous exposure to hypoxic stress (at 4 WPI) did not affect the hypoxia tolerance, which is in line with previous reports showing that salmonids are adapted to physiologically and metabolically counteract transient low oxygen levels [32,55].

The reduced hypoxia tolerance was still evident three weeks later (10 WPI) for the PRV group, but not for the PRV-H group. There was no difference in PRV RNA levels or HSMI scores between the two infected groups at this time-point. The only difference between the infected groups was the two previous hypoxic exposures of the PRV-H group, which may indicate a stress-conditioning effect [56,57]. However, this should be followed up in future studies also including non-infected fish subjected to the same hypoxic stress.

The maximum heart rate measurements at 10 WPI were in line with the reduced hypoxia tolerance observed for the PRV group. This group had lower *f*_Hmax_ compared to the PRV-H and Ctrl groups, which was significant at 19°C. Furthermore, the *T*_opt_ for aerobic scope was lower supporting that infection and/or inflammation impaired cardiac capacity. The lower *f*_Hmax_ and *T*_opt_ observed for the PRV group may be due to reflex bradycardia, which is a common cardiac response to hypoxia in fish [31], and may suggest a reduced stress tolerance at high temperatures in PRV-infected farmed Atlantic salmon. A regression analysis did not detect any impact of individual virus load (PRV Ct values) on the *f*_Hmax_ between PRV and PRV-H groups, implying that exposure to hypoxic stress rather than the viral load was explaining differences in performance.

Despite similar cardiac performance and hypoxia tolerance to the Ctrl group at 10 WPI, the PRV-H group had a reduced blood oxygen binding affinity. The improved cardiac performance and higher aerobic scope for the PRV-H group may have compensated for the reduced blood oxygen affinity. However, this remains speculative since blood oxygen affinity was not measured in the PRV group. Our results highlight the possible negative consequences of erythrocyte infection and cardiac inflammatory lesions on the cardiorespiratory performance of salmonids. The finding that short-term environmental hypoxic stress may improve tolerance to secondary acute hypoxia in post-smolts is intriguing.

The mean incipient lethal oxygen saturation measured in the current study are considerably higher than previously published for salmonids [48,58,59]. This discrepancy may be due to differences in experimental protocol, water salinity and temperature and life stage of the experimental fish [60]. The experimental fish in our study were reared in brackish water and challenged with the first HCT six weeks after transfer from freshwater. The period before and after seawater transfer is considered to be challenging and energy consuming for salmonids. The increased metabolic and physiological demands after seawater transfer may reduce the capacity to handle hypoxic environment. This may reduce the hypoxic tolerance and hence explain the higher ILOS values, as observed in our trial. Variations in hypoxia tolerance between strains of rainbow trout [48] and between individuals of Atlantic salmon [39,59] may also explain differences in observed ILOS between studies. A size or age dependent reduction in T_50_ and ILOS as the trial proceeded may be present in our study, although no firm causality between these factors has been detected in previous comparable studies [48,49,61]. Another likely explanation was the fact that the biomass increased from the first to the last HCT as the fish grew bigger. Despite minor differences in the levels of critical oxygen saturation between groups, the results strongly suggested that HSMI is associated with reduced hypoxia tolerance, reflected by the shorter time to loss of equilibrium (K-M curves for ILOS and T_50_).

Scoring of histopathological changes in heart revealed no major differences in HSMI development between the infected groups. The myocytes in the spongious layer of the salmonid heart ventricle are passively oxygenated by the venous blood entering the lumen of the ventricle. This is in contrast to the oxygenated blood from the gills supplying the compactum via the coronary circulation [31,32]. The blood flow in the coronary arteries has been shown to increase during hypoxia and exercise in rainbow trout and Coho salmon [62,63]. The subsequent effect on inflammatory development due to the difference in oxygen supply of the different myocardial layers remains to be investigated. Our findings did not detect any differences in inflammatory severity between these strata of the heart. However, this mechanism cannot be excluded on the basis of our challenge trial due to the time separating the sampling points.

At 12 WPI, when the PRV-H group had experienced three episodes of hypoxic stress, this group tended to have lower histopathology scores in the heart, indicating a better myocardial recovery for these individuals. Few studies have investigated the consequences of hypoxia on disease development following a viral infection in teleosts. In SPDV challenged Atlantic salmon post-smolts, long-term hypoxia at 12°C (60–65% O_2_ saturation for 70 days) had no effect on pancreas disease development [64]. Another study investigated the immune responses during chronic hypoxia in Atlantic salmon post-smolts stimulated with poly I:C or a water-based *Vibrio anguillarum* vaccine [65]. The fish were exposed to 58 days of hypoxia (52% O_2_ saturation at 10.5°C) and the results showed a reduced expression of immune related genes, *in vitro* and *in vivo* [65], potentially connected to hypoxia-induced production of corticosteroids such as cortisol [30,65]. Such responses may repress the innate and adaptive immune responses during viral infection and hence lower the degree of inflammation within infected tissues. In zebrafish, hypoxia induced responses have been shown to improve myocardial regeneration [66,67]. In sum, this represents putative explanations to why the PRV-H group had reduced levels of inflammation and heart lesions at 12 WPI compared to the PRV group.

Exposure to three periodic hypoxic episodes during experimental PRV-infection did not affect PRV RNA levels when compared to infected fish without hypoxic stress. Nevertheless, we cannot rule out that hypoxia during earlier stages of infection may have a different effect on virus levels. Such short-term environmental hypoxia may have effects on the integrity of barrier defense organs (i.e. skin and gill). Therefore, hypoxic stress prior to and during the actual phase of virus uptake from water should be addressed in future studies.

Measurements of Hb-oxygen affinity detected an apparent difference in P_50_ between the control group and the PRV-H group suggesting a Bohr effect occurring in the latter group. To the author’s knowledge, this is the first report showing decreased Hb-oxygen affinity in viral-infected erythrocytes in fish. Studies on rats infected with malaria (*Plasmodium berghei berghei*) showed a similar reduced Hb-oxygen affinity in infected erythrocytes at intraerythrocytic pH ≤ 7.0 [68,69], detected as a right-shift of the ODC [68]. In the latter study, ATP concentration in infected erythrocytes increased five-fold due to increased parasite production, hence explaining the reduced oxygen affinity. Soivio *et al*. [70] detected an increase in blood oxygen affinity in rainbow trout exposed to hypoxia for 12 days (25–35% O_2_, 11°C). The increase was detected within 6 h of hypoxia and reached steady state after 8 days of hypoxia. A reduced ATP content of 50% was detected in the erythrocytes of the hypoxic fish after one week [70] and was suggested to increase the oxygen carrying capacity of Hb [33]. In our study, ATP levels tended to be higher in the PRV-H group compared to non-infected controls. This corresponded to a reduction in the oxygen carrying capacity of Hb in the PRV-H group compared to the controls (right-shifted ODC, increased P_50_ and lower maximal oxygen saturation). This apparent reduced oxygen carrying capacity (sometimes referred to as the Root effect when caused by reduced intracellular pH) is a common feature of fish Hb that greatly enhances oxygen delivery under exercise or hypoxia [71]. In the present study, fish experiencing periodic hypoxia in combination with the presence of viral replication within red blood cells would likely be a compensation to increase their tissue oxygen delivery.

Interestingly, the Hb concentrations were significantly reduced in the infected groups at 4 and 7 WPI compared to the controls. The variations in Hb concentration in the Ctrl group during the trial may be due to physiological changes after seawater transfer. Furthermore, the PRV RNA levels in blood and heart correlated with Hb concentration for individuals at 7 and 10 WPI. Hemolytic anemia has been reported for a number of salmonid diseases such as viral hemorrhagic septicemia [72] and ISA [73]. Recently, a variant of PRV was demonstrated to be the causal agent of erythrocytic inclusion body syndrome (EIBS) in Coho salmon, a disease associated with anemia [13]. Although anemia is not a common finding in HSMI, the current data indicate that dips in hemoglobin may occur during the peak phase of PRV infection. However, in the linear model, PRV Ct values in blood and heart explained respectively 18 and 35% of the total variation in Hb concentration measured in PRV-infected individuals in the compiled data from 7 and 10 WPI.

The potential impact of PRV-infection and HSMI on hypoxia tolerance and cardiorespiratory performance observed in this study highlights the importance of further increasing our knowledge on this topic, in order to improve the fish health and prevent losses in Atlantic salmon aquaculture. Future research should focus on understanding the molecular and pathological mechanisms underlying the observed differences in hypoxia tolerance.

## Supporting information

S1 Table Scoring criteria for histopathological evaluation of heart sections. (PDF)

S2 Table PRV Ct values in heart and blood. Ct values are presented as range (min to max) and +/- SEM.
(PDF)

S3 Table Table of number of fish sampled in the different groups and at specific time-points. Ctrl: non-infected controls, PRV: PRV-infected fish, PRV-H: PRV-infected fish exposed to periodic hypoxic stress.
(PDF)

S1 Fig Weight of the experimental fish. All fish included in each group from Day 0 to 15 weeks post infection. Ctrl: non-infected controls, PRV: PRV-infected fish, PRV-H: PRV-infected fish exposed to periodic hypoxic stress. The lower and upper border of boxes indicates the 25^th^ and 75^th^ percentiles, respectively and the centerline indicates the 50^th^ percentile. The upper and lower whiskers correspond to the highest and lowest value of the 1.5*IQR (inter-quartile range). Significance is indicated by * with a *p* < 0.05.
(TIF)

S2 Fig K-factor of all fish in every group from Day 0 to 15 weeks post infection. Ctrl: non-infected controls, PRV: PRV-infected fish, PRV-H: PRV-infected fish exposed to periodic hypoxic stress. The lower and upper border of boxes indicates the 25^th^ and 75^th^ percentiles, respectively and the centerline indicates the 50^th^ percentile. The upper and lower whiskers correspond to the highest and lowest value of the 1.5*IQR (inter-quartile range). Significance is indicated by * with a *p* < 0.05.
(TIF)

S3 Fig Relation between weight and oxygen saturation at ILOS. A. Weight of the individuals included in the hypoxia challenge test (HCT). Ctrl: non-infected controls, PRV: PRV-infected fish, PRV-H: PRV-infected fish exposed to periodic hypoxic stress. The lower and upper border of boxes indicates the 25^th^ and 75^th^ percentiles, respectively and the centerline indicates the 50^th^ percentile. The upper and lower whiskers correspond to the highest and lowest value of the 1.5*IQR (inter-quartile range). Mean weight (+/-SEM) for each group at each time-point is stated in the text box. Significance is indicated by * with a *p* < 0.05. B. Individual weight plotted against the respective oxygen saturation at ILOS for all HCT’s; 4 (grey dots), 7 (black dots) and 10 (red dots) weeks post infection (WPI) is indicated. The output of a linear regression and Pearson correlation analysis is stated. C. Individual weight plotted against the respective oxygen saturation at ILOS for all HCT’s; Ctrl (grey dots), PRV (black dots) and PRV-H (red dots) groups are indicated. The output of the same linear regression analysis as performed in B is stated. D. Table of output from the linear regression and Pearson correlation analysis for each group and separate HCTs performed at 4, 7 and 10 WPI. “lm(weight ~ O_2_%)” indicate the linear regression model used.
(TIF)

S4 Fig Time-course of water oxygenation and temperature for each HCT. (TIF)

S5 Fig Histological scoring of inflammatory changes in the heart–Heart rate. Development of inflammatory changes is displayed for fish included in the heart rate measurement at 10 weeks post infection (WPI). Ctrl: non-infected controls, PRV: PRV infected fish, PRV-H: PRV infected fish exposed to periodic hypoxic stress. Inflammatory changes in epicardium, compactum and spongiosum were scored from sections of the heart ventricle using a continuous visual analogue scale ranging from 0–3. The total HSMI score was calculated from the mean of scores from the separate heart compartments. The lower and upper border of boxes indicates the 25^th^ and 75^th^ percentiles, respectively and the centerline indicates the 50^th^ percentile. The upper and lower whiskers correspond to the highest and lowest value of the 1.5*IQR (inter-quartile range). A non-parametric Mann-Whitney unpaired rank test was performed between the groups and no statistical differences were detected (*p* > 0.05).
(TIF)

S6 Fig Pearson correlation analysis between PRV Ct values and heart rate. (TIF)

S7 Fig ATP levels in every group at Day 0 and at 4 and 10 weeks post infection. Ctrl: non-infected controls, PRV: PRV-infected fish, PRV-H: PRV-infected fish exposed to periodic hypoxic stress. The lower and upper border of boxes indicates the 25^th^ and 75^th^ percentiles, respectively and the centerline indicates the 50^th^ percentile. The upper and lower whiskers correspond to the highest and lowest value of the 1.5*IQR (inter-quartile range). Significance is indicated by + with a *p* = 0.07.
(TIF)

S8 Fig Pearson correlation analysis and linear regression analysis of PRV Ct values and hemoglobin (Hb) concentrations in fish sampled for disease development. The analysis was performed on merged data-points from 7 and 10 weeks post infection. Correlation and linear regression output between Hb concentrations and PRV Ct values in blood (A) and heart (B).
(TIF)

S1 File Dataset. (XLSX)
