# Supplementary material for: Hypoxia tolerance and responses to hypoxic stress during heart and skeletal muscle inflammation in Atlantic salmon (Salmo salar)
Source: PLoS One. 2017 Jul 11;12(7):e0181109. doi: 10.1371/journal.pone.0181109 (PMC5507449; doi:10.1371/journal.pone.0181109)
Supplement: S1 Table — (PDF) [file pone.0181109.s001.pdf]

Score	Criteria
**Epicarditis**	
0	No pathological changes observed
0.1 – 1.0	Focal or multifocal mononuclear infiltration of one cell layer in epicardium
1.1 – 2.0	Multifocal, moderate infiltration of 2-3 layers of inflammatory cells in epicardium
2.1 – 3.0	Extensive infiltration of >3 layers of inflammatory cells in epicardium
**Myocardial inflammation in the compactum**	
0	No pathological changes observed
0.1 – 1.0	Focal, mild infiltration of a limited number of mononuclear inflammatory cells
1.1 – 2.0	Focal to multifocal, moderate infiltration of mononuclear inflammatory cells
2.1 – 3.0	Difuse and extensive infiltration of mononuclear inflammatory cells and myocardial necrosis
**Myocardial inflammation in the spongiosum**	
0	No pathological changes observed
0.1 – 1.0	Focal, mild infiltration of a limited number of mononuclear inflammatory cells
1.1 – 2.0	Focal to multifocal, moderate infiltration of mononuclear inflammatory cells
2.1 – 3.0	Difuse and extensive infiltration of mononuclear inflammatory cells and myocardial necrosis
