# Supplementary material for: Hypoxia tolerance and responses to hypoxic stress during heart and skeletal muscle inflammation in Atlantic salmon (Salmo salar)
Source: PLoS One. 2017 Jul 11;12(7):e0181109. doi: 10.1371/journal.pone.0181109 (PMC5507449; doi:10.1371/journal.pone.0181109)
Supplement: S3 Table — Ctrl: non-infected controls, PRV: PRV-infected fish, PRV-H: PRV-infected fish exposed to periodic hypoxic stress. (PDF) [file pone.0181109.s003.pdf]

	WPI	0	3	4		7			10			12			15		
		Day 0	PRV	PRV	Ctrl	PRV-H	PRV	Ctrl	PRV-H	PRV	Ctrl	PRV-H	PRV	Ctrl	PRV-H	PRV	Ctrl
Sampling for disease developement	Blood (heperanized)	12	12	20	10	10	10	10	10	10	8	10	10	10	20	20	20
	Heart (RNAlater)	12	12	20	10	10	10	10	10	10	10	10	10	10	20	20	20
	Heart (Formalin)	8	0	20	10	10	10	10	10	10	10	10	10	10	10	10	10
Hypoxia challenge test	Blood			20	10	20	20	17	20	20	10						
	Heart (RNAlater)								19	19							
	Heart (Formalin)					20	20	20	19	20	20						
Heart rate measurement	Blood (heperanized)								16	14	10						
	Heart (Formalin)								16	16	16						
Hemoglobin oxygen dissociation curve	Blood (heperanized)								12		6						
