# Supplementary figures and images for: Hypoxia tolerance and responses to hypoxic stress during heart and skeletal muscle inflammation in Atlantic salmon (Salmo salar)
Source: PLoS One. 2017 Jul 11;12(7):e0181109. doi: 10.1371/journal.pone.0181109 (PMC5507449; doi:10.1371/journal.pone.0181109)

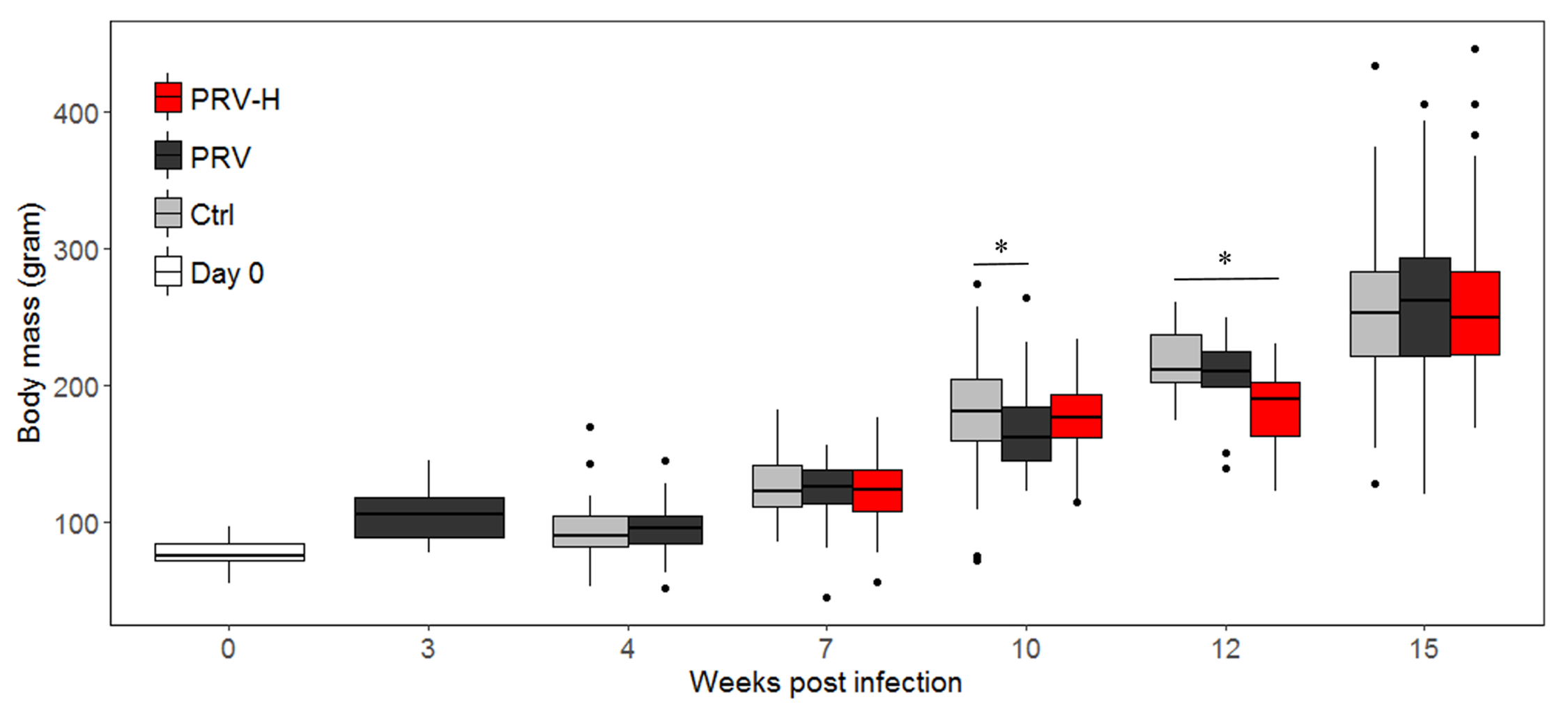

Supplement: S1 Fig — All fish included in each group from Day 0 to 15 weeks post infection. Ctrl: non-infected controls, PRV: PRV-infected fish, PRV-H: PRV-infected fish exposed to periodic hypoxic stress. The lower and upper border of boxes indicates the 25th and 75th percentiles, respectively and the centerline indicates the 50th percentile. The upper and lower whiskers correspond to the highest and lowest value of the 1.5*IQR (inter-quartile range). Significance is indicated by * with a p < 0.05. (TIF) [file pone.0181109.s004.tif]

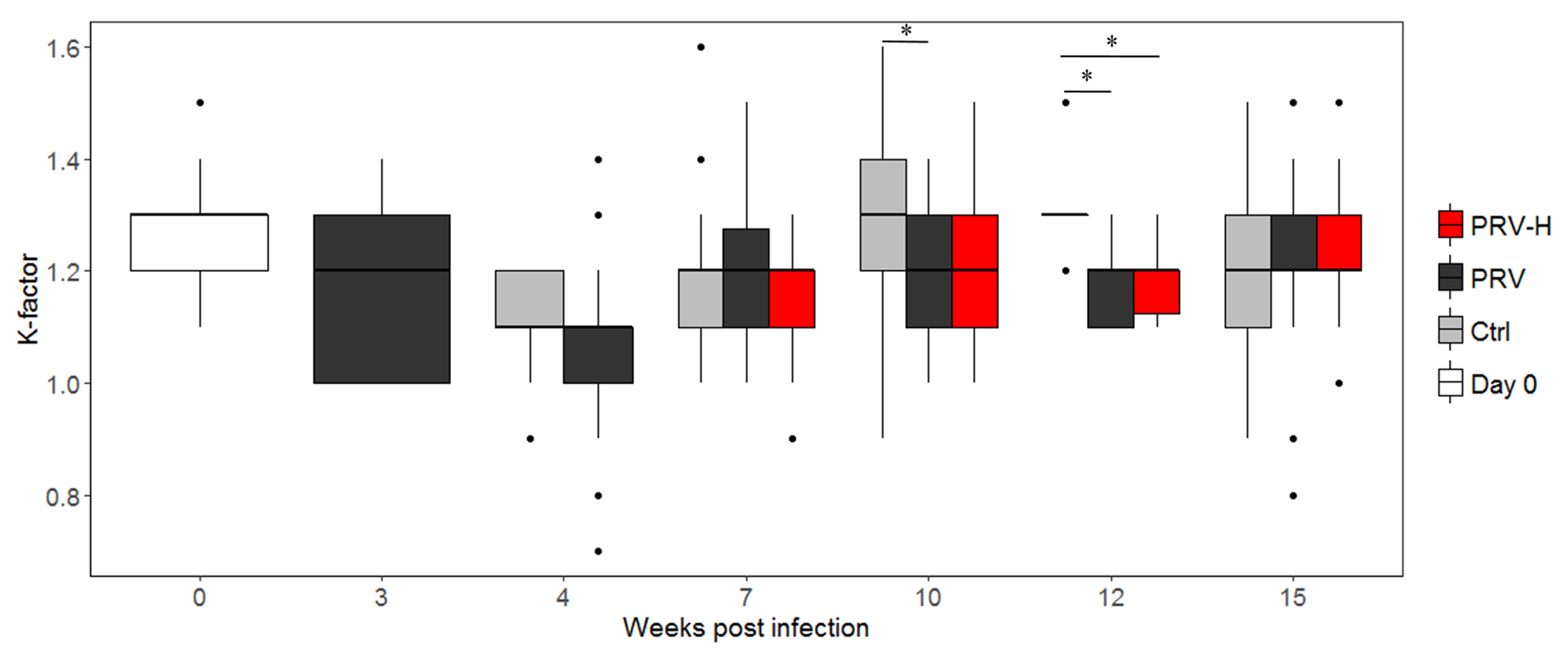

Supplement: S2 Fig — Ctrl: non-infected controls, PRV: PRV-infected fish, PRV-H: PRV-infected fish exposed to periodic hypoxic stress. The lower and upper border of boxes indicates the 25th and 75th percentiles, respectively and the centerline indicates the 50th percentile. The upper and lower whiskers correspond to the highest and lowest value of the 1.5*IQR (inter-quartile range). Significance is indicated by * with a p < 0.05. (TIF) [file pone.0181109.s005.tif]

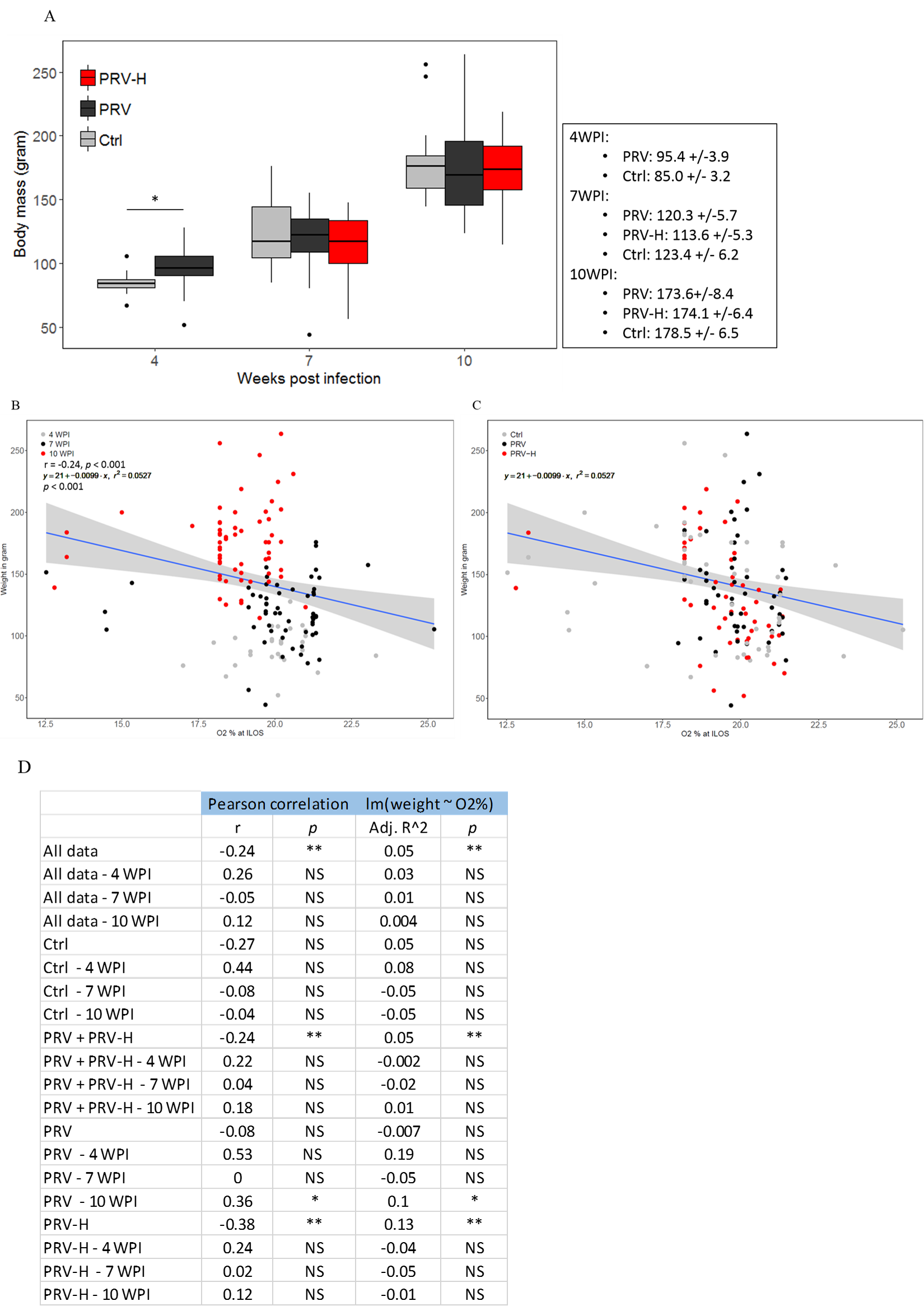

Supplement: S3 Fig — A. Weight of the individuals included in the hypoxia challenge test (HCT). Ctrl: non-infected controls, PRV: PRV-infected fish, PRV-H: PRV-infected fish exposed to periodic hypoxic stress. The lower and upper border of boxes indicates the 25th and 75th percentiles, respectively and the centerline indicates the 50th percentile. The upper and lower whiskers correspond to the highest and lowest value of the 1.5*IQR (inter-quartile range). Mean weight (+/-SEM) for each group at each time-point is stated in the text box. Significance is indicated by * with a p < 0.05. B. Individual weight plotted against the respective oxygen saturation at ILOS for all HCT’s; 4 (grey dots), 7 (black dots) and 10 (red dots) weeks post infection (WPI) is indicated. The output of a linear regression and Pearson correlation analysis is stated. C. Individual weight plotted against the respective oxygen saturation at ILOS for all HCT’s; Ctrl (grey dots), PRV (black dots) and PRV-H (red dots) groups are indicated. The output of the same linear regression analysis as performed in B is stated. D. Table of output from the linear regression and Pearson correlation analysis for each group and separate HCTs performed at 4, 7 and 10 WPI. “lm(weight ~ O2%)” indicate the linear regression model used. (TIF) [file pone.0181109.s006.tif]

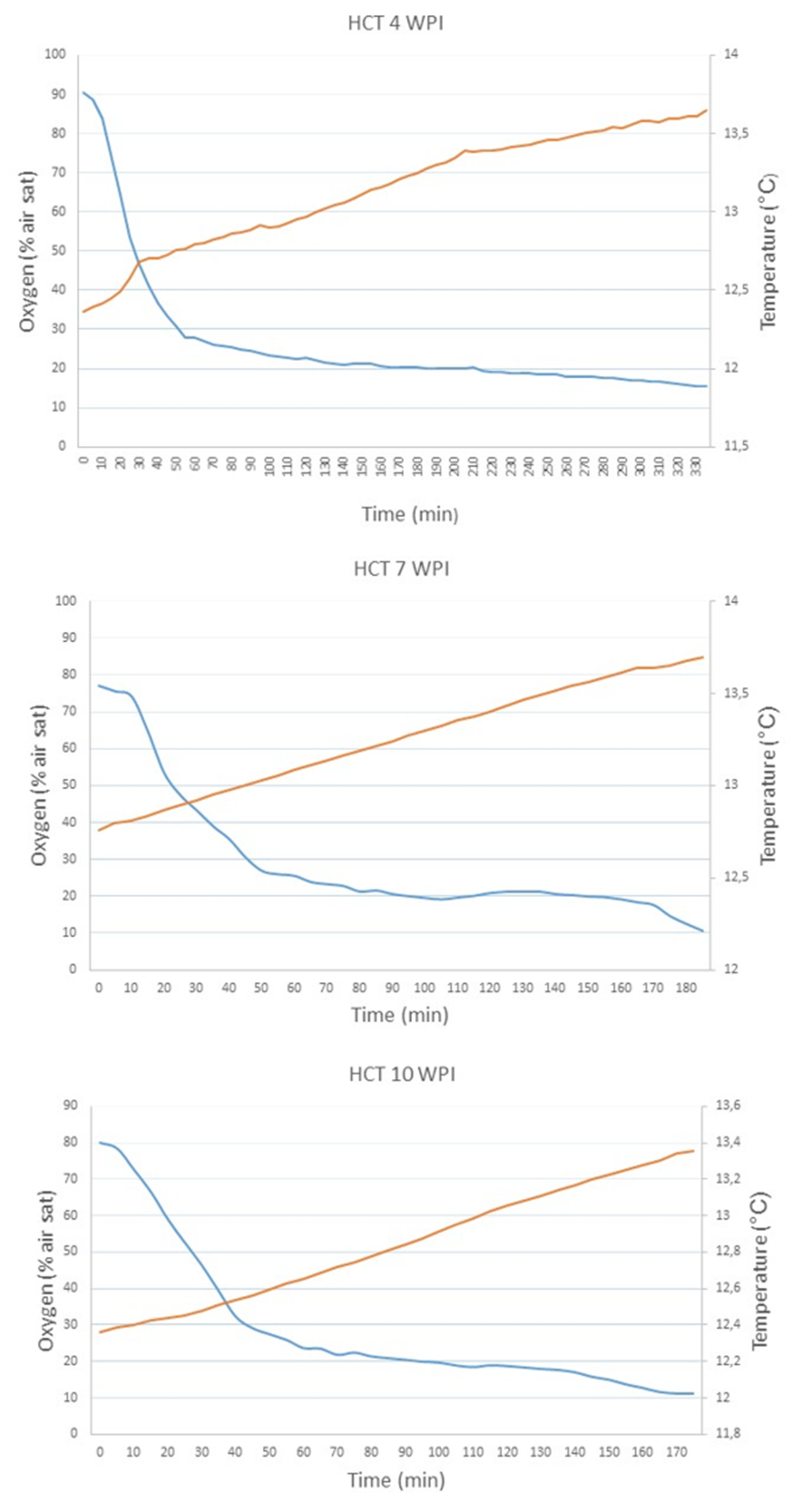

Supplement: S4 Fig — (TIF) [file pone.0181109.s007.tif]

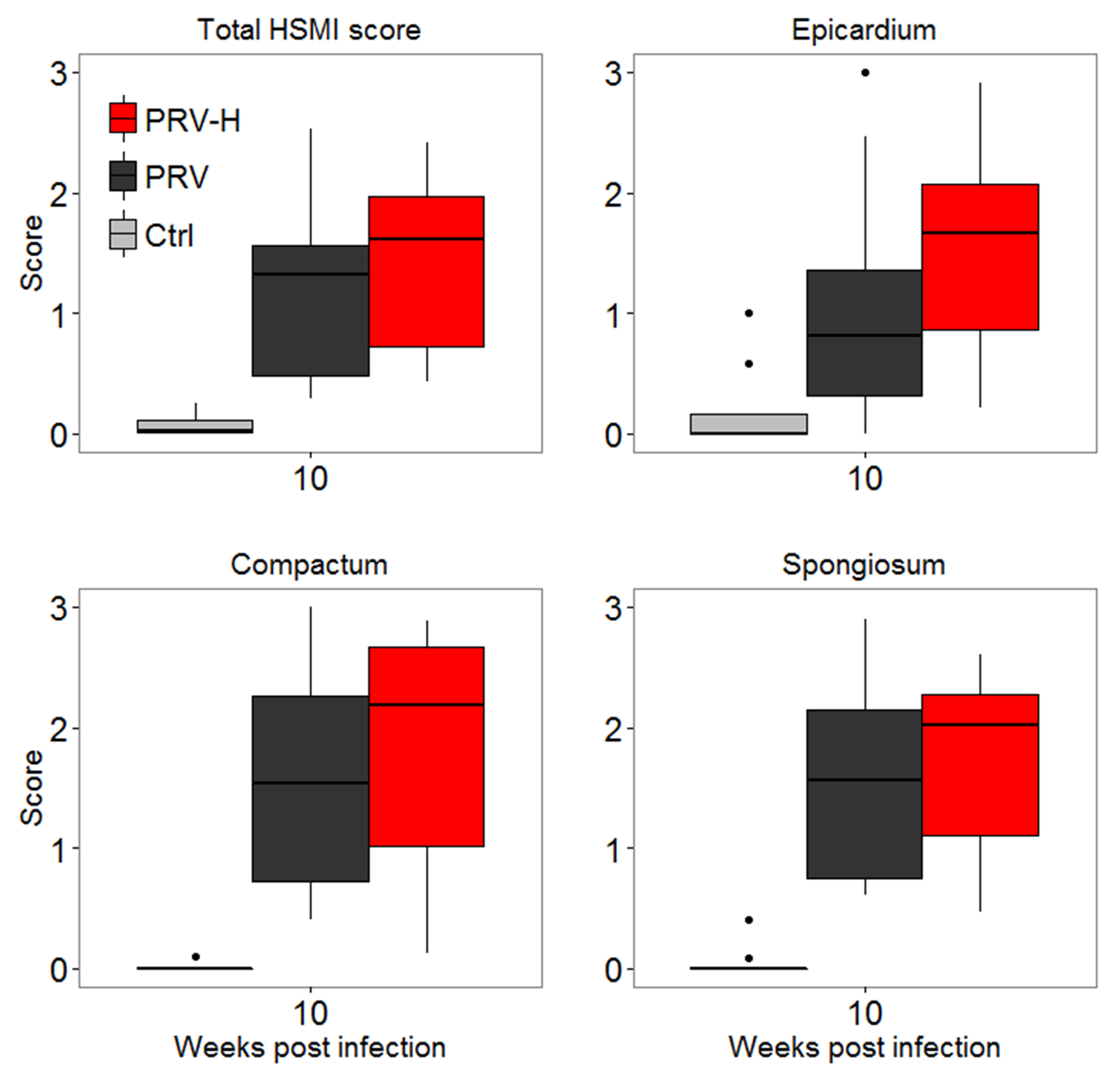

Supplement: S5 Fig — Development of inflammatory changes is displayed for fish included in the heart rate measurement at 10 weeks post infection (WPI). Ctrl: non-infected controls, PRV: PRV infected fish, PRV-H: PRV infected fish exposed to periodic hypoxic stress. Inflammatory changes in epicardium, compactum and spongiosum were scored from sections of the heart ventricle using a continuous visual analogue scale ranging from 0–3. The total HSMI score was calculated from the mean of scores from the separate heart compartments. The lower and upper border of boxes indicates the 25th and 75th percentiles, respectively and the centerline indicates the 50th percentile. The upper and lower whiskers correspond to the highest and lowest value of the 1.5*IQR (inter-quartile range). A non-parametric Mann-Whitney unpaired rank test was performed between the groups and no statistical differences were detected (p > 0.05). (TIF) [file pone.0181109.s008.tif]

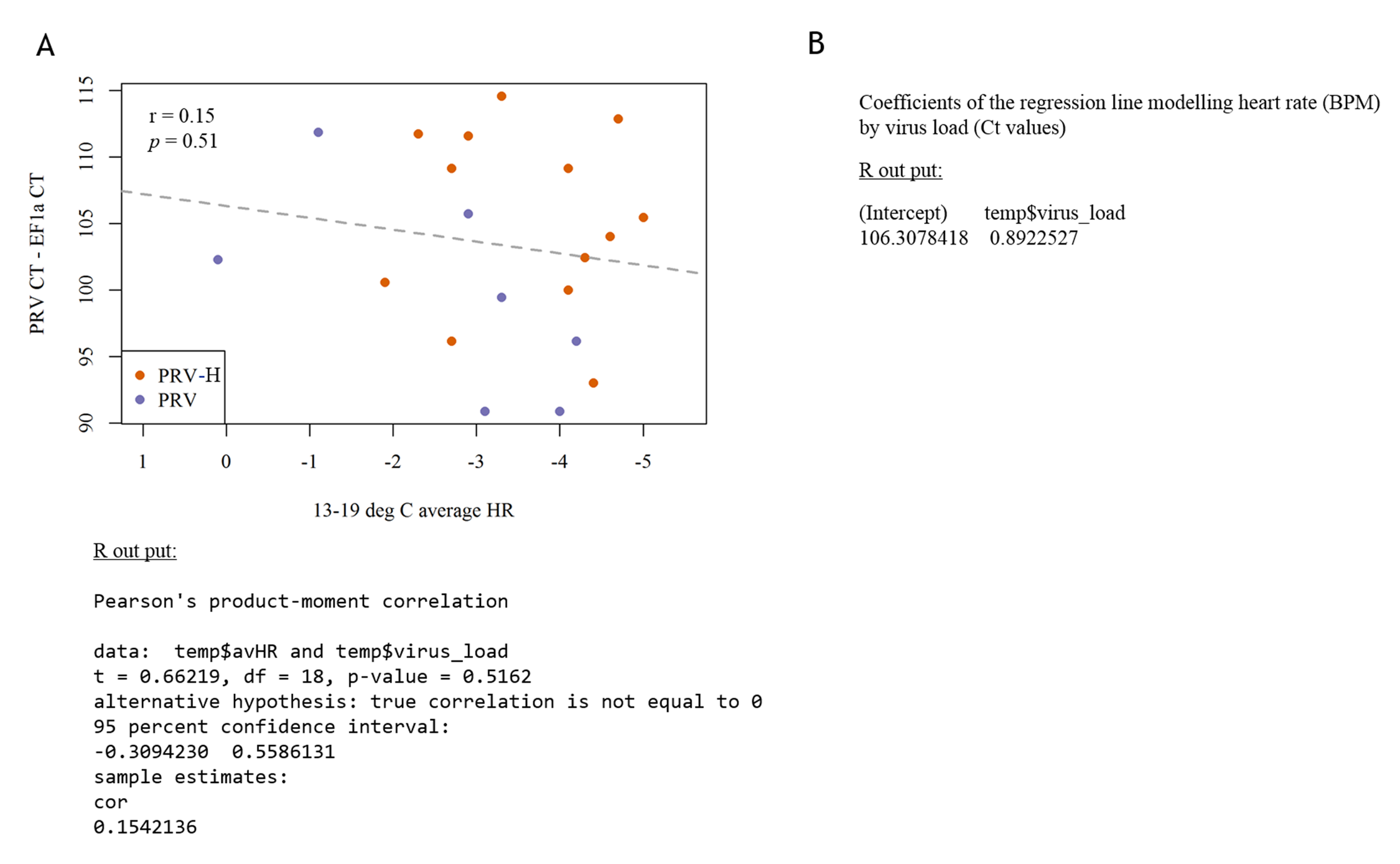

Supplement: S6 Fig — (TIF) [file pone.0181109.s009.tif]

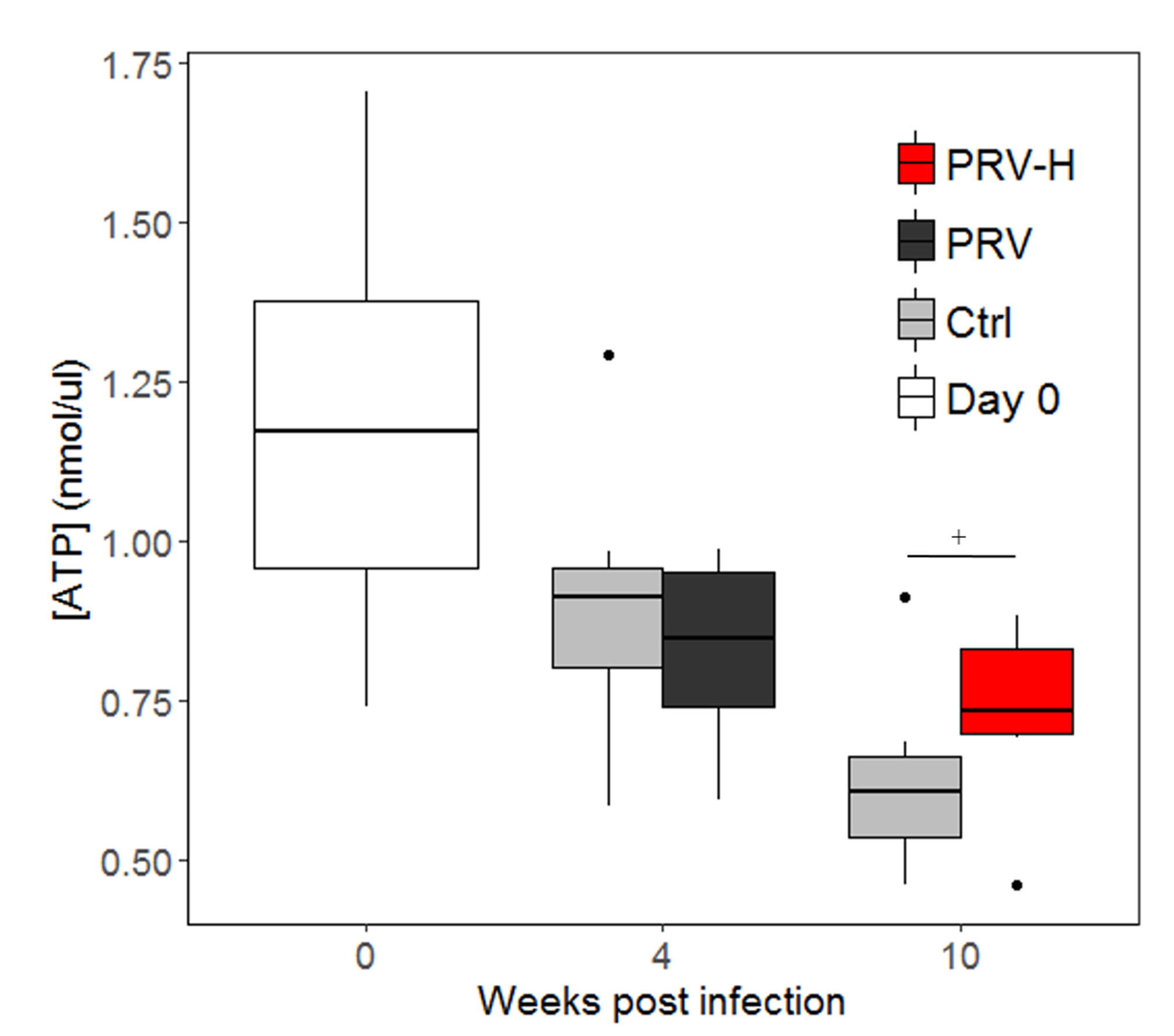

Supplement: S7 Fig — Ctrl: non-infected controls, PRV: PRV-infected fish, PRV-H: PRV-infected fish exposed to periodic hypoxic stress. The lower and upper border of boxes indicates the 25th and 75th percentiles, respectively and the centerline indicates the 50th percentile. The upper and lower whiskers correspond to the highest and lowest value of the 1.5*IQR (inter-quartile range). Significance is indicated by + with a p = 0.07. (TIF) [file pone.0181109.s010.tif]

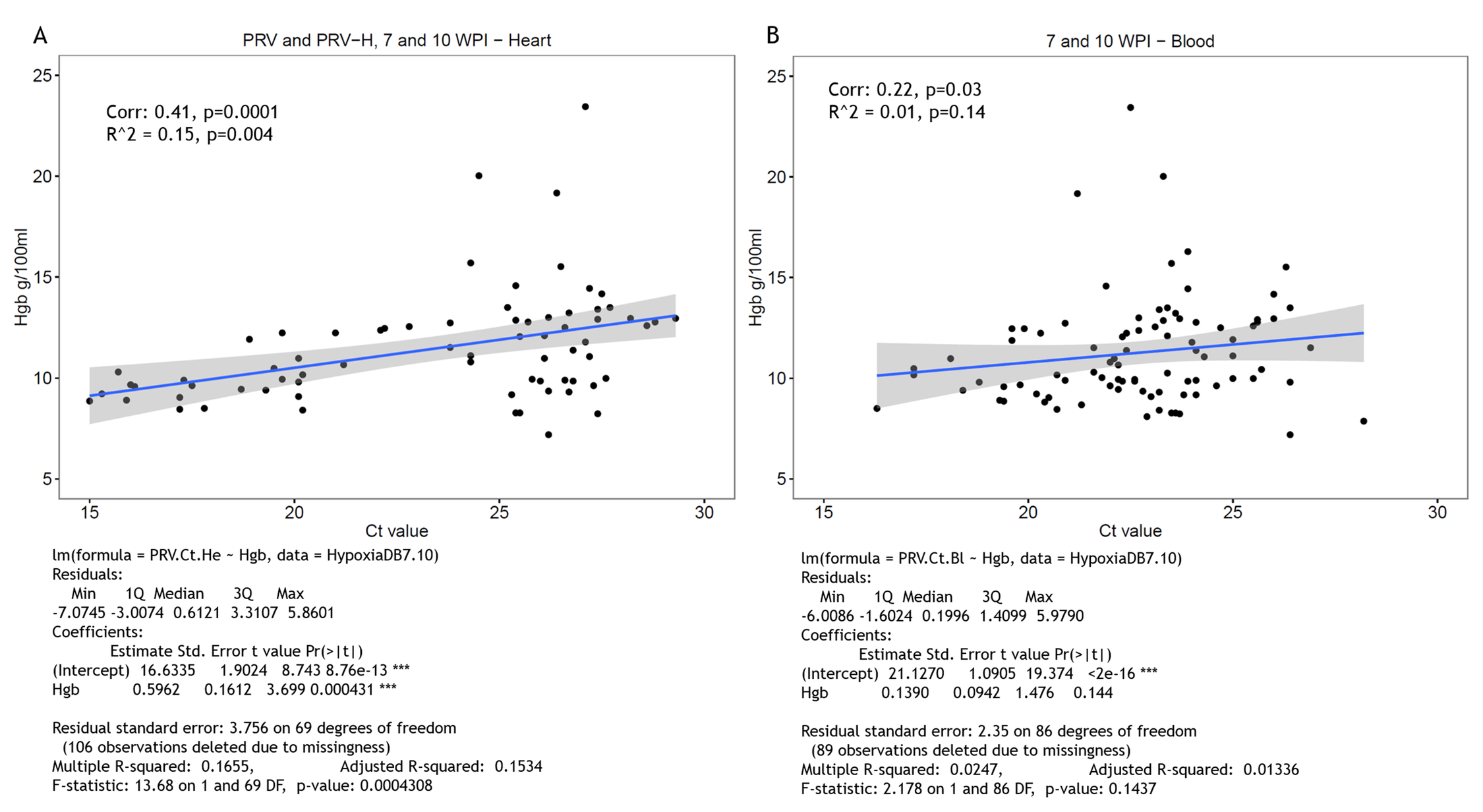

Supplement: S8 Fig — The analysis was performed on merged data-points from 7 and 10 weeks post infection. Correlation and linear regression output between Hb concentrations and PRV Ct values in blood (A) and heart (B). (TIF) [file pone.0181109.s011.tif]
